# High-Content Small Molecule Screen Identifies a Novel Compound That Restores AP-4-Dependent Protein Trafficking in Neuronal Models of AP-4-Associated Hereditary Spastic Paraplegia

**DOI:** 10.21203/rs.3.rs-3036166/v1

**Published:** 2023-06-12

**Authors:** Afshin Saffari, Barbara Brechmann, Cedric Boeger, Wardiya Afshar Saber, Hellen jumo, Dosh Whye, Delaney Wood, Lara Wahlster, Julian Alecu, Marvin Ziegler, Marlene Scheffold, Kellen Winden, Jed Hubbs, Elizabeth Buttermore, Lee Barrett, Georg Borner, Alexandra Davies, Mustafa Sahin, Darius Ebrahimi-Fakhari

**Affiliations:** Boston Children’s Hospital, Harvard Medical School; Boston Children’s Hospital, Harvard Medical School; Boston Children’s Hospital, Harvard Medical School; Boston Children’s Hospital/Harvard Medical School; Boston Children’s Hospital, Harvard Medical School; Boston Children’s Hospital, Harvard Medical School; Boston Children’s Hospital, Harvard Medical School; Boston Children’s Hospital, Harvard Medical School; Harvard Medical School; Boston Children’s Hospital, Harvard Medical School; Boston Children’s Hospital; Boston Children’s Hospital; Boston Children’s Hospital; Boston Children’s Hospital; Max Planck Institute of Biochemistry; Max Planck Institute of Biochemistry; Boston Children’s Hospital; Boston Children’s Hospital, Harvard Medical School

## Abstract

Unbiased phenotypic screens in patient-relevant disease models offer the potential to detect novel therapeutic targets for rare diseases. In this study, we developed a high-throughput screening assay to identify molecules that correct aberrant protein trafficking in adaptor protein complex 4 (AP-4) deficiency, a rare but prototypical form of childhood-onset hereditary spastic paraplegia, characterized by mislocalization of the autophagy protein ATG9A. Using high-content microscopy and an automated image analysis pipeline, we screened a diversity library of 28,864 small molecules and identified a lead compound, *C*-*01*, that restored ATG9A pathology in multiple disease models, including patient-derived fibroblasts and induced pluripotent stem cell-derived neurons. We used multiparametric orthogonal strategies and integrated transcriptomic and proteomic approaches to delineate putative molecular targets of *C*-*01* and potential mechanisms of action. Our results define molecular regulators of intracellular ATG9A trafficking and characterize a lead compound for the treatment of AP-4 deficiency, providing important proof-of-concept data for future Investigational New Drug (IND)-enabling studies.

## INTRODUCTION

Despite remarkable advances in our ability to delineate the genetic causes of rare neurological diseases, it is estimated that specific therapies exist for less than 5% ^1^. Thus, there is a significant unmet need for developing and implementing novel platforms for drug discovery. Informed by disease-relevant cellular phenotypes, automated and unbiased cell-based high-throughput small molecule screens have the potential to uncover novel therapeutic targets ^2, 3, 4, 5, 6^.

Adaptor protein complex 4 (AP-4)-related hereditary spastic paraplegia (AP-4-HSP), which comprises *AP4B1*-associated SPG47 (OMIM #614066), *AP4M1*-associated SPG50 (OMIM #612936), *AP4E1*-associated SPG51 (OMIM #613744) and *AP4S1*-associated SPG52 (OMIM #614067), is a rare but prototypical form of childhood-onset complex hereditary spastic paraplegia (HSP) and an important genetic mimic of cerebral palsy ^7, 8^. Children with AP-4-HSP present with features of both a neurodevelopmental disorder (e.g., early-onset global developmental delay and seizures, microcephaly, and developmental brain malformations) and a neurodegenerative disease (e.g., progressive spasticity and weakness, loss of ambulation, and extrapyramidal movement disorders) ^7, 8, 9, 10^. AP-4-HSP is caused by bi-allelic loss-of-function variants in any of the four AP-4 subunits (ε, β4, μ4, σ4), leading to impaired AP-4 assembly and function ^11, 12, 13, 14, 15^. AP-4 is an obligate heterotetrameric protein complex ^16, 17, 18^ that mediates transport from the trans-Golgi network (TGN) to the cell periphery, including sites of autophagosome biogenesis ^19, 20^. Three independent groups identified the core autophagy protein and lipid scramblase ATG9A as a major cargo of AP-4 ^11, 12, 13, 14, 21^, linking loss of AP-4 function to defective autophagy ^22, 23^. AP-4 deficiency in non-neuronal ^11, 12, 21, 24, 25^ and neuronal cells ^13, 14, 15^ leads to an accumulation of ATG9A in the TGN, including in iPSC-derived neurons from AP-4-HSP patients ^15^. From this body of work, and overlapping neuronal phenotypes of AP-4 ^13, 14, 26, 27^ and *Atg9a*^28^ knockout mice, the following working model for AP-4 deficiency emerges: (1) AP-4 is required for trafficking of ATG9A from the TGN; (2) loss-of-function variants in AP-4 subunits lead to a loss of AP-4 function; (3) ATG9A accumulates in the TGN leading to a reduction of axonal delivery of ATG9A; (4) lack of ATG9A at the distal axon impairs autophagy leading to axonal degeneration. Other AP-4 cargo proteins identified to date include the poorly characterized transmembrane proteins SERINC1 and SERINC3 ^12^, and the endocannabinoid producing enzyme DAG lipase beta (DAGLB) ^29^.

In this study, we leverage intracellular ATG9A mislocalization as a cellular readout for AP-4 deficiency to develop a large-scale, automated, multiparametric, unbiased phenotypic small molecule screen for modulators of ATG9A trafficking in patient-derived cellular models. We employed this platform to screen a diversity library of 28,864 novel small molecules in AP-4-deficient patient fibroblasts and identified 503 compounds that re-distribute ATG9A from the TGN to the cytoplasm. Through a series of orthogonal assays in neuronal cells, including differentiated *AP4B1*^*KO*^ SH-SY5Y cells and human induced pluripotent stem cell (hiPSC)-derived neurons from AP4-HSP patients, we defined a series of 5 novel compounds that restore neuronal phenotypes of AP-4-deficiency. In a comprehensive multiparametric analysis, a novel small molecule, termed *C*-*01*, emerged as a lead compound with an EC50 of ~ 5μM. Target deconvolution strategies using transcriptomic and proteomic profiling revealed that *C*-*01* modulates intracellular vesicle trafficking and increases autophagic flux, potentially through differential expression of several RAB (Ras-associated binding) proteins.

Our findings demonstrate the ability of carefully designed high-throughput screens to identify molecular targets for AP-4 deficiency and support the development of *C*-*01* as a novel therapeutic for AP-4-HSP.

## RESULTS

### Primary screening of 28,864 compounds in fibroblasts from AP-4-HSP patients identifies 503 active compounds

**Figure 1:**
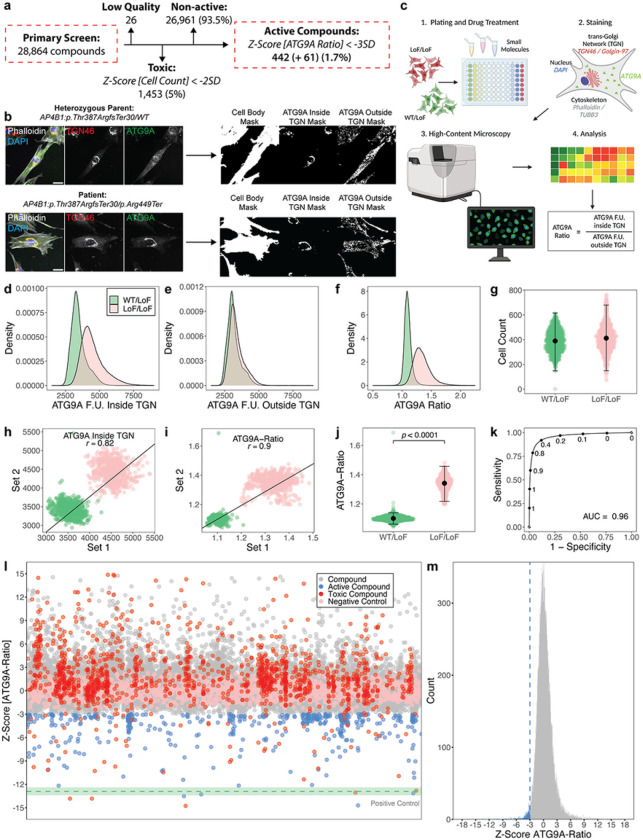
Establishment of a cell-based phenotypic small molecule screening platform using ATG9A translocation as a surrogate for AP-4 function and primary screening of 28,864 novel small molecule compounds. (a) Overview of the primary screening of 28,864 novel small molecule compounds in fibroblasts from a patient with AP-4-HSP due to biallelic loss-of-function variants in *AP4B1*. (b) Illustration of the automated image analysis pipeline. Representative images of fibroblasts from a patient with HSP-*SPG47* (negative control, LoF/LoF) and their sex-matched heterozygous parent (positive control, WT/LoF) are shown. Four markers are captured including Phalloidin (grey), DAPI (blue), TGN (red) and ATG9A (green). The TGN and ATG9A channels are additionally depicted in greyscale. Through a series of masks, the intracellular distribution of ATG9A is calculated at the level of individuals cells, with hundreds of thousands to millions of cells per experiment. Scale bar: 20μm. (c) Overview of the high-throughput platform and workflow. The assay is miniaturized to 96- or 384-well microplates. Cells are stained using automated liquid handlers and imaged using an automated high-content confocal microscope, followed by automated image analysis. Primary metric is the ‘ATG9A ratio’, which is calculated by dividing the ATG9A fluorescence intensity (F.U.) inside the TGN by the ATG9A fluorescence intensity in the cytoplasm. (d-f) The distribution of ATG9A fluorescence intensities inside (d) and outside (e) the TGN, as well as the ATG9A ratio (f) are shown on a per cell basis. 99,927 WT/LoF and 119,522 LoF/LoF cells were quantified. (g) Cell counts are measured for each experimental well. 1312 wells were analyzed per condition. (h&i) Replicate plots were generated by random sampling of the 82 plates from the primary screen in two groups. Similar positions on the assay plates were plotted against each other with respect to the ATG9A fluorescence intensity inside the TGN (h) and the ATG9A ratio (i). Replicate correlations for both analysis strategies were assessed by averaging the Pearson correlation coefficients of 100 random sampling tests. The ATG9A ratio shows a mean Pearson correlation coefficient (*r*) of 0.9, while the ATG9A fluorescence inside the TGN shows an average r of 0.82. (j) To demonstrate the discriminative power of the ATG9A ratio in separating positive and negative controls, statistical testing was done using the Mann-Whitney U test. Quantification was done using per well means. 1312 wells per condition were included. Positive and negative controls showed a robust separation (*p* < 0.0001). (k) To test the robustness of separation of the ATG9A ratio between positive (WT/LoF) and negative controls (LoF/LoF), a dataset containing measurement for 99,927 WT/LoF and 119,522 LoF/LoF cells was partitioned into a training set (70% of data) and a test set (30%). A generalized linear model was trained using the training set. The performance of the prediction model using the test set is shown in (k). The AUC is 0.96. (l) Impact of 28,864 compounds applied for 24h at a concentration 10μM. Z-scores for the primary metric, the ATG9A ratio, are shown. All data points represent per well means. The mean of the positive control (WT/LoF) is shown as a green dotted line. The green shaded areas represent ± 1 SD. Active compounds were *a priori* defined as those reducing the ATG9A ratio by at least 3 SD compared to negative controls. Toxicity was defined as a reduction of cell count of at least 2SD compared to the negative control. 501 compounds show activity by reducing the ATG9A ratio by more than 3 SD. (m) Distribution of Z-scores of all non-toxic 27,412 compounds. Active compounds are highlighted in blue.

A diversity library of 28,864 novel small molecules was provided by Astellas Pharma Inc. and arrayed in 384-well microplates. The primary screen was conducted in fibroblasts from a well-characterized patient with core clinical features of SPG47 ^8^ and bi-allelic loss-of-function variants in *AP4B1* (NM_001253852.3: c.1160_1161del (p.Thr387ArgfsTer30) / c.1345A > T (p.Arg449Ter)) (Fig. 1a,b). Fibroblasts from the sex-matched parent (unaffected heterozygous carrier) served as controls. The assay was fully automated, miniaturized to 384-well microplates, and compounds were added for 24h at a single concentration of 10μM (Fig. 1c).

The ATG9A ratio (ATG9A fluorescence intensity inside the TGN vs. in the cytoplasm) was used as the primary assay metric, as established previously ^15, 21^. The population distributions of the subcellular ATG9A signal inside and outside the TGN, at the level of single cells for negative (bi-allelic loss-of-function, LoF/LoF) and positive (heterozygous carriers, WT/LoF) controls are shown in Fig. 1d and 1e. ATG9A ratios demonstrated symmetrical and approximately normal distributions and robust separation of both groups (Fig. 1f). Cell counts were similar for positive and negative controls, excluding cell death or changes in proliferation rates as possible confounding factors (Fig. 1g). To test for reproducibility across replicates, assay plates were randomly sampled into two sets, and similar positions on the assay plates were plotted against each other (Fig. 1h,i). Random sampling was simulated 100 times, and mean correlation coefficients were calculated. Using the ATG9A ratio (Fig. 1i) as a primary readout resulted in higher replicate correlation (mean *r* = 0.90 ± 0.002 SD), compared to absolute ATG9A intensities (Fig. 1h) (mean *r* = 0.82 ± 0.0008 SD). ATG9A ratios showed robust discriminative power between positive and negative controls (LoF/LoF mean: 1.1 ± 0.02 SD, n = 1312 wells vs. WT/LoF mean: 1.34 ± 0.05 SD, n = 1312 wells; Mann-Whitney U test, *p* < 0.0001) (Fig. 1j). The ATG9A ratio as the primary outcome metric was further supported by a generalized linear model, which demonstrated high specificity and sensitivity (Fig. 1k, AUC: 0.96). Source data for assay performance are provided in Supplementary File 1.

Throughout the screen, assay performance was monitored using established quality control metrics for cell-based screens (Z’ robust ≥ 0.3, strictly standardized median difference ≥ 3, and an inter-assay coefficient of variation ≤ 10%) ^30, 31, 32^. All assay metrics were calculated for positive and negative controls of the same assay plate to avoid bias by inter-plate variability. Predefined thresholds were met by all assay plates (Supplementary Fig. 1a and Supplementary File 2). The results of the primary screen are summarized in Fig. 1l and 1m, and the complete dataset is provided in Supplementary File 3.

Of the 28,838 compounds, 26 were excluded due to non-quantifiable ATG9A signal, exceptionally low cell counts or imaging artifacts. The remaining 28,812 compounds were evaluated for changes in cell count and the ATG9A ratio. The vast majority (n = 26,961, 93.5%) did not show any significant reduction in the ATG9A ratio (defined as a reduction by at least 3 SD). 1,435 (5.0%) compounds were excluded due to toxicity, defined as a reduction in the mean cell count by at least 2 SD compared to the negative controls. Only a small subset of 503 compounds (1.7%) reduced the ATG9A ratio by 3 or more SD compared to negative controls (Fig. 1m). Of these, 61 (0.2%) also reduced cell counts, while the remaining 442 (1.5%) showed no toxicity.

In summary, from this high-throughput primary screen, 503 active compounds were identified and selected for further testing.

### Counter-screen in fibroblasts from AP-4-HSP patients confirms 16 compounds that lead to a dose-dependent redistribution of ATG9A

**Figure 2:**
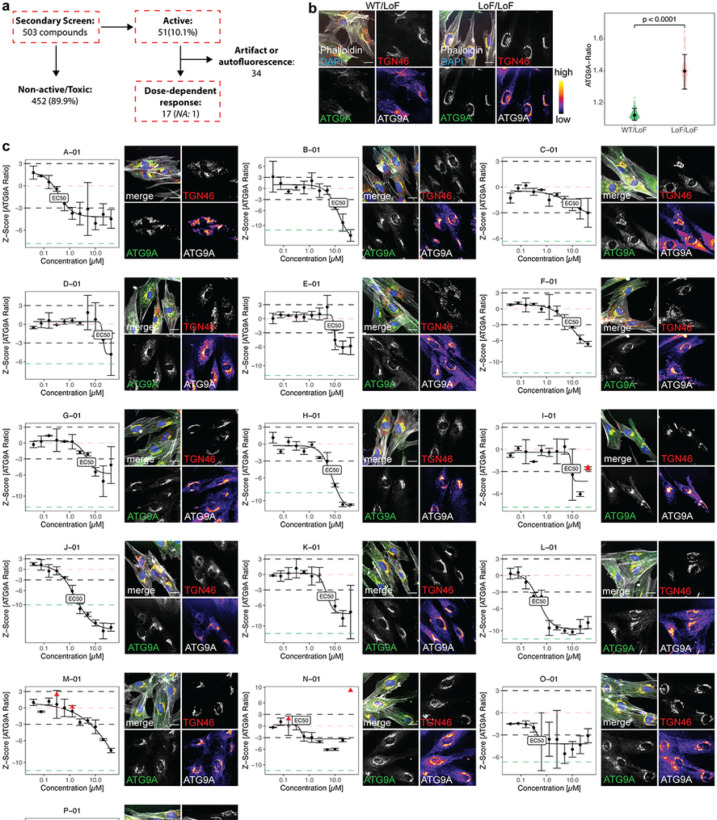
Counter-screen in fibroblasts from AP-4-HSP patients confirms 16 compounds that lead to dose-dependent redistribution of ATG9A. (a) Overview of the counter-screen of the 503 active compounds identified in the primary screen. To assess for dose-dependent effects, compounds were screened in AP-4-HSP patient-derived fibroblasts in 384-well microplates using 11-point titrations ranging from 40nM to 40μM. All concentrations were screened in duplicates. Active compounds were *a priori* defined as those reducing the ATG9A ratio by at least 3SD compared to negative controls, in more than one concentration. Toxicity was defined as a reduction of the cell count of at least 2 SD compared to negative controls. 51 compounds demonstrated a clear and reproducible dose-response relationship and raised no suspicion for autofluorescence on automated and manual review. 34 compounds showed autofluorescence or resulted in imaging artifacts. One active compound was unavailable from the manufacturer and was therefore excluded from subsequent testing. (b) Baseline differences in the ATG9A distribution in WT/LoF (n=269) vs. LoF/LoF (n=269) fibroblasts. Statistical testing was done using the Mann-Whitney U test. Positive and negative controls showed a robust separation (p < 0.0001). (c) Dose-response curves were fitted using a four-parameter logistic regression model, and EC50 concentrations were calculated. All concentrations were tested in biologic duplicates. Most EC50 were in the low micromolar range (median: 4.66mM, IQR: 8.63). Black dashed lines represent the *a priori* defined thresholds of +/− 3SD compared to the negative control (LoF/LoF). Red triangles represent toxic concentrations based on the *a priori* defined threshold of a reduction of cell counts of at least 2 SD compared to the negative control. The salmon-colored dashed line represents the mean of negative controls, while the green-colored dashed line depicts the mean of the positive controls (WT/LoF). Representative images of the EC50 are shown for each active compound. Representative images show a merge of the 4 channels: Phalloidin (grey), DAPI (blue), TGN (red) and ATG9A (green), as well as the TGN and ATG9A channels in greyscale. For better illustration of differences in ATG9A signals, the fluorescence intensities of the ATG9A channel are additionally shown using a color lookup table. Scale bar: 20μm. *NA*: not available

To validate the 503 active compounds identified in the primary screen, compounds were retested for dose-dependency using an 11-point dose range (range: 40nM to 40μM) (Fig. 2a). Source data for the secondary screen are provided in Supplementary File 4. All concentrations were screened in biological duplicates and subjected to the same quality control metrics as in the primary screen (Supplementary Fig. 1b and Supplementary File 5). Similar to the results from the primary screen, ATG9A ratios for negative and positive controls showed a robust separation (LoF/LoF mean: 1.4 ± 0.07 (SD), n = 269 wells, vs. WT/LoF mean: 1.12 ± 0.02 (SD), n = 269 wells, Mann-Whitney U test, *p* < 0.0001, Fig. 2b). Activity in the secondary screen was defined as the ability to reduce the ATG9A ratio by at least 3 SD in both replicates and at least 2 different concentrations, without exerting toxicity. 51 compounds (10.1%) met these *a priori* defined criteria (Supplementary Fig. 2a,b). After manually verifying image quality and validating dose-response relationships, compounds were triaged (Fig. 2a and Supplementary Fig. 2a,b). Seventeen compounds demonstrated a clear and reproducible dose-response relationship, without evidence of image artifacts or autofluorescence. The EC50 for most compounds were in the low micromolar range (median: 4.66μM, IQR: 8.63, Fig. 2). 34 compounds were found to carry autofluorescence or imaging artifacts and were thus excluded from further testing (Supplementary Fig. 2b). One active compound was unavailable from the manufacturer and was removed.

In summary, a counter-screen in AP-4-deficient patient fibroblasts confirmed and established dose-dependent effects on intracellular ATG9A distribution for 16 compounds (Fig. 2c).

### Orthogonal assays in neuronal models of AP-4-deficiency confirm 5 active compounds

**Figure 3:**
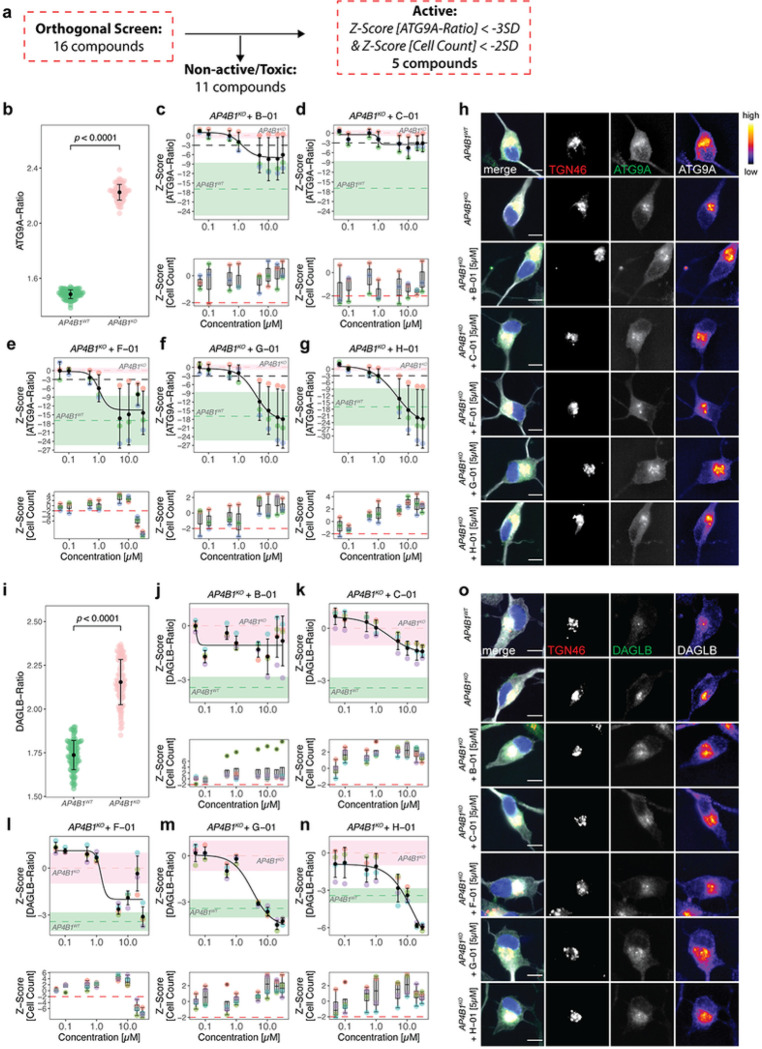
Orthogonal assays in *AP4B1*^*KO*^ SH-SY5Y cells confirm 5 active compounds. (a) Overview of the orthogonal screen of 16 active compounds in differentiated *AP4B1*^*KO*^ SH-SY5Y cells, a neuronal model of AP-4 deficiency. Active compounds were *a priori* defined as those reducing the ATG9A ratio by at least 3 SD compared to negative controls. Toxicity was defined as a reduction of cell count of at least 2 SD compared to the negative control. (b) Baseline differences in ATG9A ratios of *AP4B1*^*WT*^ vs. *AP4B1*^*KO*^ SH-SY5Y cells were quantified from 160 *AB4B1*^*WT*^ and 158 *AB4B1*^*KO*^ wells from 5 assay plates. Statistical testing was performed using the Mann-Whitney U test. Positive and negative controls showed a robust separation (*p* < 0.0001). (c-g) Dose-response curves for ATG9A ratios in *AB4B1*^*KO*^ cells treated with different compounds. Data points represent per-well means from 3 different assay plates. Dashed lines show mean Z-scores for positive (green) and negative (salmon) controls. Shaded areas represent ± 1 SD. (h) Representative images of the intracellular ATG9A distribution for individual compounds. The merged image shows beta-3 tubulin (grey), DAPI (blue), the TGN (red) and ATG9A (green). The TGN and ATG9A channels are further separately depicted in greyscale. Scale bar: 10μm. (i) Baseline differences of DAGLB ratios in *AP4B1*^*WT*^ vs. *AP4B1*^*KO*^ cells were quantified from 192 *AB4B1*^*WT*^ and 192 *AB4B1*^*KO*^ wells from 4 assay plates. Statistical testing was done using the Mann-Whitney U test. Positive and negative controls showed a robust separation (*p* < 0.0001). (j-n) Dose-response curves for DAGLB ratios in *AB4B1*^*KO*^ cells treated with different compounds. All data points represent per-well means from 4 different assay plates. Dashed lines show mean Z-scores for positive (green) and negative (salmon) controls. Shaded areas represent ± 1 SD. (o) Representative images of the intracellular DAGLB distribution for individual compounds. The merge shows beta-3 tubulin (grey), DAPI (blue), the TGN (red) and DAGLB (green). The TGN and DAGLB channels are further separately depicted in greyscale. Scale bar: 10μm.

To validate active compounds from the secondary screen in a human cell line with neuron-like properties, the ATG9A assay was optimized for neuroblastoma-derived SH-SY5Y cells following a 5-day neuronal differentiation protocol with retinoic acid ^33^ (Fig. 3a). SH-SY5Y cells with stable expression of a *AP4B1*-targeting CRISPR/Cas9 machinery (*AP4B1*^*KO*^) ^12^ served as negative controls while AP4B1-wildtype (*AP4B1*^*WT*^) cells were used as positive controls. All 16 active compounds were tested in an 8-point dose range (50nM to 30μM) with a treatment duration of 24h. Quantification of the ATG9A ratio in differentiated SH-SY5Y cells showed a robust separation between control conditions (*AP4B1*^*KO*^: 1.80 ± 0.06 (SD), n = 158 wells vs. *AP4B1*^*WT*^: 1.17 ± 0.03 (SD), n = 160 wells, Mann-Whitney U test, *p* < 0.0001, Fig. 3b, Supplementary File 6). Compounds were evaluated based on their dose-dependent reduction of the ATG9A ratio and absence of cell toxicity. Eleven of 16 compounds were excluded due to lacking activity (n = 7), suspicion for artefacts or autofluorescence (n = 3), or obvious changes in cellular morphology (n = 1) (Supplementary Fig. 3). Of the five remaining compounds, three restored the ATG9A ratio to levels of wildtype controls (*F-01*, *G-01* and *H-01*) while two compounds (*B-01* and *C-01*) led to a reduction by at least 3 SD at higher concentrations (Fig. 3c–h).

To assess whether these effects were specific to ATG9A or similar effects were also present for other AP-4 cargo proteins, we turned to a second neuronal AP-4 cargo protein, DAGLB ^29^. Similar to ATG9A, the DAGLB ratio (DAGLB fluorescence intensity in the TGN vs. in the cytoplasm) showed a robust separation between *AP4B1*^*WT*^ and *AP4B1*^*KO*^ cells (*AP4B1*^*KO*^: 1.80 ± 0.1 (SD), n = 192 wells vs. *AP4B1*^*WT*^: 1.36 ± 0.07 (SD), n = 192 wells, Mann-Whitney U test, *p* < 0.0001, Fig. 3i, Supplementary File 6). All five active compounds showed activity in the DAGLB assay, suggesting a broader effect on the trafficking of at least 2 AP-4 cargo proteins from the TGN. Again, *F-01*, *G-01* and *H-01* (Fig. 3l–o) resulted in normalization of the intracellular DAGLB distribution, while *B-01* and *C-01* led to a moderate reduction of DAGLB ratios at higher concentrations (Fig. 3j,k,o).

**Figure 4:**
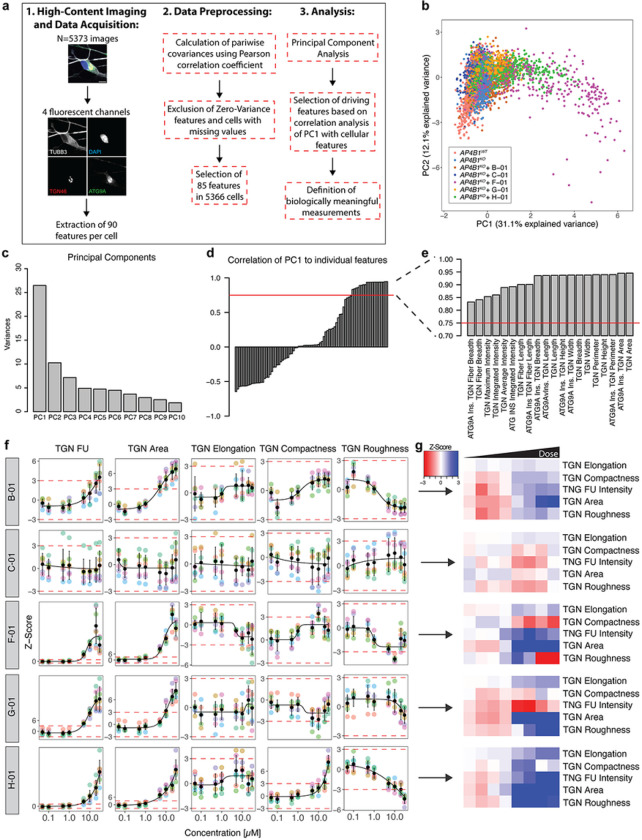
Multiparametric profiling of 5 active compounds in *AP4B1*^*KO*^ SH-SY5Y cells. (a) Multiparametric profiling of images of 5373 cells were acquired using 4 fluorescent channels. Scale: 10μm. A total of 90 measurements per cell were generated for the cytoskeleton (beta-3 tubulin), the nucleus (DAPI), the TGN (TNG46) and ATG9A vesicles (ATG9A). The different steps of data preprocessing and phenotypic clustering using principal component analysis (PCA) are shown. (b) PCA shows different clusters of cells based on 85 phenotypic features. Experimental conditions are color-coded. The first two principal components (PC1 and PC2) explain 43.2% of the observed variance. (c) Bar plot summarizing the variance explained by the first 10 principal components (PCs). Most of the variance is explained by PC1 and to a lesser degree PC2. (d) Correlation analysis of PC1 with all 85 features using the Pearson correlation coefficient. Red dashed lines represent cut-offs for correlations >0.75. (e) Zoom-in on selected features of interest showing a correlation with PC1 >0.75. (f) Measurements of TGN intensity and descriptors of TGN shape and network complexity for the individual hit compounds as line graphs and (g) summarized using heatmap visualization.

Since small molecules can have pleotropic effects on cellular functions and organellar morphology, we adapted a multiparametric morphological profiling approach ^34^. Eighty-five measurements of the nucleus, cytoskeleton, global cell morphology, the TGN, and ATG9A vesicles were automatically computed for each image, serving as a rich and unbiased source for interrogating biological perturbations induced by compound treatment (Supplementary File 6). Principal component analysis was used to reduce dimensionality and cluster images based on their properties (Fig. 4a and Supplementary Fig. 4). Positive and negative controls clustered closely together and were separated only by the ATG9A signal (Fig. 4b and Supplementary Fig. 4a). *B-01*, *C-01* and *G-01* showed properties comparable to positive and negative controls, suggesting little off-target effects (Fig. 4b Supplementary Fig. 4b,c,e). *F-01* and *H-01*, however, changed cellular morphology in a dose-dependent manner (Fig. 4b and Supplementary Fig. 4d,f), with changes mainly driven by the first principal component, accounting for 31.1% of the observed variance (Fig. 4c). To decipher the phenotypic alterations responsible for these changes, the Pearson correlation coefficients of the first principal component with each measurement were calculated (Fig. 4d). Features with a correlation coefficient > 0.75 were selected to define morphological profiles (Fig. 4e). Interestingly, TGN fluorescence intensity and morphology seemed to be the most significant drivers for the separation, suggesting that disruption of TGN integrity potentially biased the assessment of ATG9A ratios in cells treated with compounds *F-01* and *H-01* (Fig. 4b and Supplementary Fig. 4d,f).

Following these analyses, TGN fluorescence intensity and morphological measures such as TGN area and elongation, as well as compactness and roughness, as indicators of the complexity of the TGN, were quantified for cells treated with all five active compounds (Fig. 4f,g). While *C-01* showed stable TGN signal and morphology across all assessed measurements, the other compounds depicted some degree of change. Again, *F-01* and *H-01* seemed to result in TGN changes in a dose-dependent manner while *B-01* and *G-01* led to only moderate alterations (Fig. 4f,g). Of note, these changes to TGN morphology were not detectable by visual inspection but only delineated through an automated analysis of ~ 600 images containing ~ 30,000 cells per group, showcasing the power of our automated, unbiased, high-throughput platform.

### C-01 restores ATG9A and DAGLB trafficking in hiPSC-derived neurons from AP-4-HSP patients

**Figure 5:**
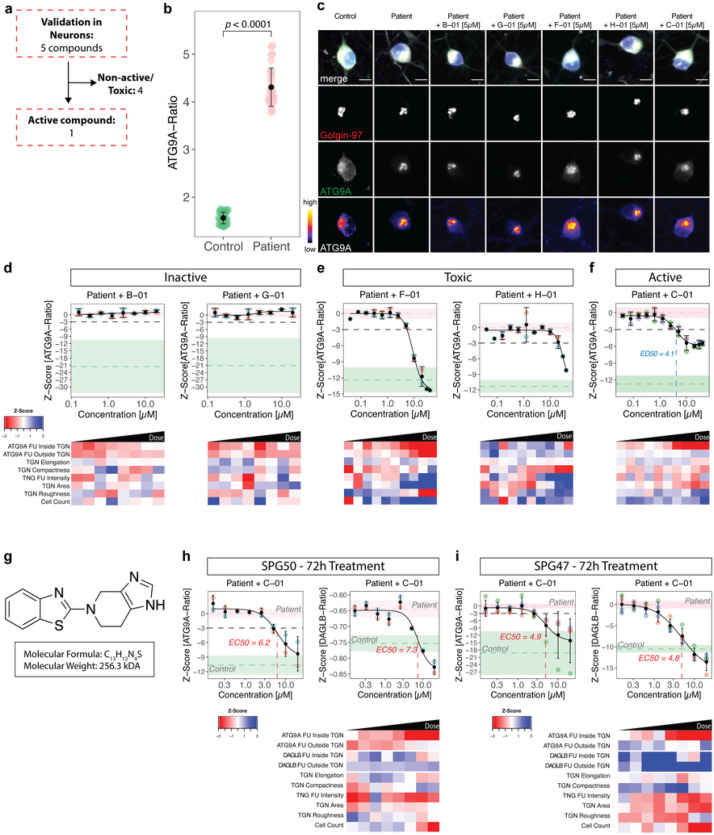
Compound *C-01* restores ATG9A and DAGLB trafficking in iPSC-derived neurons from AP-4-HSP patients. (a) Overview of the testing of 5 active compounds in iPSC-derived cortical neurons from a patient with *AP4M1*-associated SPG50 compared to heterozygous controls (same-sex parent). Active compounds were defined as those reducing the ATG9A ratio by at least 3 SD compared to negative controls (patient-derived iPSC-neurons treated with vehicle). Toxicity was defined as a reduction of cell count of at least 2 SD compared to the negative control. (b) Baseline differences of ATG9A ratios in controls vs. patient-derived iPSC-neurons were quantified using per well means of 60 wells per condition from 5 plates. Statistical testing was done using the Mann-Whitney U test. Positive and negative controls showed a robust separation (*p* < 0.0001). (c) Representative images of iPSC-neurons from a patient with SPG50 treated with individual compounds at 5μM for 24h (~EC50 in prior experiments). The merge shows beta-3 tubulin (grey), DAPI (blue), the Golgi (red) and ATG9A (green). The Golgi and ATG9A channels are further separately depicted in greyscale. For better illustration of differences in ATG9A signals, the fluorescence intensities of the ATG9A channel are additionally shown using a color lookup table. Scale: 10μm. (d-f) Dose-response curves for ATG9A ratios in iPSC-neurons from a patient with SPG50 treated with individual compounds for 24h, along with their morphological profiles depicted as heatmaps. All data points represent per-well means of 3–4 independent differentiations. Dashed lines show mean Z-scores for positive (green) and negative (salmon) controls. Shaded areas represent ± 1SD. (g) Chemical synthesis and structure of lead compound *C-01*. (h&i) Dose-response curves for ATG9A and DAGLB ratios in iPSC-neurons from a patient with SPG50 (h) and an additional patient with SPG47 (i) after prolonged treatment with *C-01*for 72h, along with the morphologic profile depicting changes in cellular ATG9A and DAGLB distribution, TGN intensity and morphology and cell count. All data points represent per-well means of 2 independent differentiations. Dashed lines show mean Z-scores for positive (green) and negative (salmon) controls. Shaded areas represent ± 1 SD.

Informed by the findings in differentiated *AP4B1*^*KO*^ SH-SY5Y cells, we next investigated whether these results would translate to human neurons. hiPSCs from patients with AP-4-HSP due to biallelic loss-of-function variants in *AP4M1* (NM_004722.4: c.916C > T (p.Arg306Ter) / c.694dupG (p.Glu232GlyfsTer21)) and *AP4B1* (NM_001253852.3: c.1160_1161del (p.Thr387ArgfsTer30) / c.1345A > T (p.Arg449Ter)) were generated ^35, 36^ and differentiated into glutamatergic cortical neurons using established protocols ^15, 37, 38^. hiPSC-derived neurons from sex-matched parents (unaffected heterozygous carriers) served as controls (Fig. 5a and Supplementary File 7). Baseline quantification of ATG9A ratios in DIV (day *in vitro*) 14 neurons treated with vehicle for 24h showed robust separation between patient and control lines, exceeding the differences observed in AP-4-deficient fibroblasts and differentiated SH-SY5Y cells (SPG50 patient mean: 4.31 ± 0.4 (SD), n = 60 wells vs. heterozygous control: 1.56 ± 0.12 (SD), n = 60 wells, Mann-Whitney U test, *p* < 0.0001, Fig. 5b). Neurons were treated for 24h in 8-point dose titration experiments. *B-01* and *G-01* lacked activity on the ATG9A ratio and were thus excluded (Fig. 5d). *C-01, F-01* and *H-01*, by contrast, showed a robust reduction in the ATG9A ratio (Fig. 5e,f). A multiparametric analysis showed that, similar to observations in *AP4B1*^*KO*^ SH-SY5Y cells, only *C-01* preserved TGN integrity (Fig. 5f), while *F-01* and *H-01* impacted TGN morphology, suggesting off-target effects (Fig. 5e). Based on its favorable profile, *C-01* was selected as a lead compound and was re-synthesized for further testing (Fig. 5g). Prolonged treatment of *C-01* for 72h to test for ATG9A and DAGLB translocation, demonstrated that *C-01* was able to restore ratios of both AP-4 cargo proteins to levels close to controls with an EC50 of ~ 5μM, while maintaining a favorable profile (Fig. 5h, Supplementary File 7). This greater effect on ATG9A distribution, compared to the ~ 50% reduction of the ATG9A ratio at 24h treatment, suggests a time- and dose-dependent effect. *C-01* changed the ATG9A ratio through simultaneously decreasing ATG9A intensities inside the TGN and increasing cytoplasmic ATG9A levels, suggesting ATG9A translocation as the most likely mechanism of action. No changes in TGN morphology or any other cellular measurements were observed, indicating overall preservation of cellular morphology and little off-target effects. A similar pattern was observed with respect to DAGLB translocation (Fig. 5h). These findings were confirmed in a second set of experiments in hiPSC-derived neurons from a patient with SPG47 (Fig. 5i, Supplementary File 7), demonstrating that findings extend to other forms of AP-4-deficiency.

Taken together, *C-01* emerged as a robust modulator of ATG9A and DAGLB trafficking in human neurons from patients with AP-4 deficiency.

### Target deconvolution using transcriptomic and proteomic analyses delineates putative mechanisms of action for C-01

To explore potential mechanisms of action of *C-01* in an unbiased manner, we used a multi-omics approach, combining bulk RNA sequencing and unbiased label-free quantitative proteomics (source data are provided in Supplementary Files 8–10).

**Figure 6:**
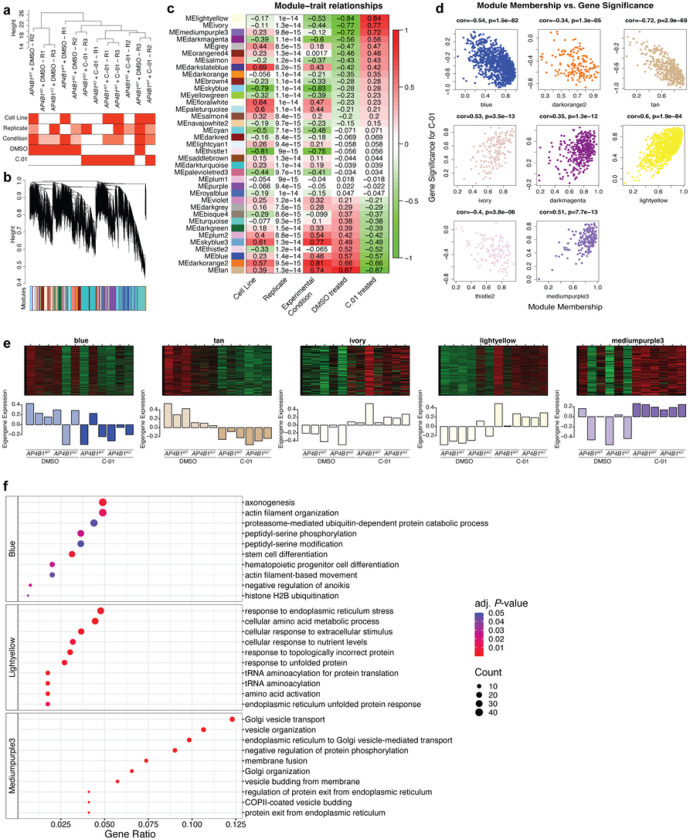
Target deconvolution using bulk RNA sequencing and weighted gene co-expression network analysis in *AP4B1*^*KO*^ SH-SY5Y cells treated with *C-01*. (a) Hierarchical clustering of 12 samples using average linkage showed two main clusters based on treatment with vehicle vs. *C-01*, irrespective of cell line. (b) Cluster dendrogram of 18,506 expressed genes based on topological overlap. Clusters of co-expressed genes (“modules”) were isolated using hierarchical clustering and adaptive branch pruning. (c) Heatmap visualization of the correlation of gene expression profiles (“module eigengene”, ME) of each module with measured traits. Pearson correlation coefficients are shown for each cell of the heatmap. (d) Intramodular analysis of module membership (MM) and gene significance (GS) for highly correlated modules, allowing identification of genes that have high significance with treatment as well as high connectivity to their modules. (e) ME expression profiles for the top 5 co-expressed modules. (f) Gene ontology enrichment analysis showed enriched pathways in 3/5 modules. Pathways were considered differentially expressed with an FDR < 0.05.

First, RNA sequencing was conducted in differentiated *AP4B1*^*WT*^ and *AP4B1*^*KO*^ SH-SY5Y cells treated for 72h with either vehicle or compound *C-01* (5μM, Supplementary File 8). Analysis of differential gene expression identified few significant transcriptional changes in response to *C-01* treatment, suggesting that this compound does not elicit major alterations in gene expression or induce many off-target effects (Supplementary Fig. 5). Since changes in gene expression caused by short-duration small molecule treatments might not reach predefined cut-offs for standard differential expression analyses, and because compounds might affect groups of genes in shared pathways rather that modifying single target genes, we adapted an unbiased and unsupervised network approach to identify groups of co-expressed genes. Hierarchical clustering of samples showed that treatment with *C-01*, regardless of cell line, was the main differentiator in our dataset (Fig. 6a). To identify the gene networks responsible for these changes, weighted gene co-expression network analysis (WGCNA) ^39, 40^ was used to group the 18,506 expressed genes into 36 co-expression modules (Fig. 6b). Gene expression profiles within each module were summarized using the “module eigengene” (ME), defined as the first principal component (PC) of a module ^41^. Within each module, the association of MEs with measured traits were examined by correlation analysis (Fig. 6c). Eight modules that showed an absolute correlation coefficient > 0.5 were selected for further evaluation. For these selected modules, ME based connectivity was determined for every gene by calculating the absolute value of the Pearson correlation between the expression of the gene and the respective ME, producing a quantitative measure of module membership (MM). Similarly, the correlation of individual genes with *C-01* treatment was computed, defining gene significance (GS) for *C-01*. Using the GS and MM, an intramodular analysis was performed, allowing identification of genes that have high significance with treatment as well as high connectivity to their modules (Fig. 6d). Five modules were significantly related to *C-01* treatment, defined as showing an absolute correlation coefficient between MM and GS > 0.5 (Fig. 6e). A list of the genes contained in each module along with their module membership is provided in Supplementary File 9. To summarize the biological information contained in these modules of interest, gene ontology (GO) analysis was performed, which demonstrated enrichment in biological pathways in three out of the five assessed modules (Fig. 6f). The ‘blue module’ showed down-regulation of pathways involved in axonogenesis, actin filament organization and proteasome-mediated pathways. The ‘light-yellow module’ contained genes involved in ER stress response, amino acid metabolism and transcription. Finally, the ‘mediumpurple3 module’ depicted upregulation of genes involved in vesicular transport, particularly involving TGN and ER-associated transport, as well as membrane and vesicle dynamics. This last module showed the highest gene ratios (defined as the percentage of total differentially expressed genes in the given GO term) and lowest *P*-values of all differentially regulated pathways across all modules, suggesting the upregulation of alternative vesicle mediated transport mechanisms by compound *C-01* (Fig. 6f).

**Figure 7:**
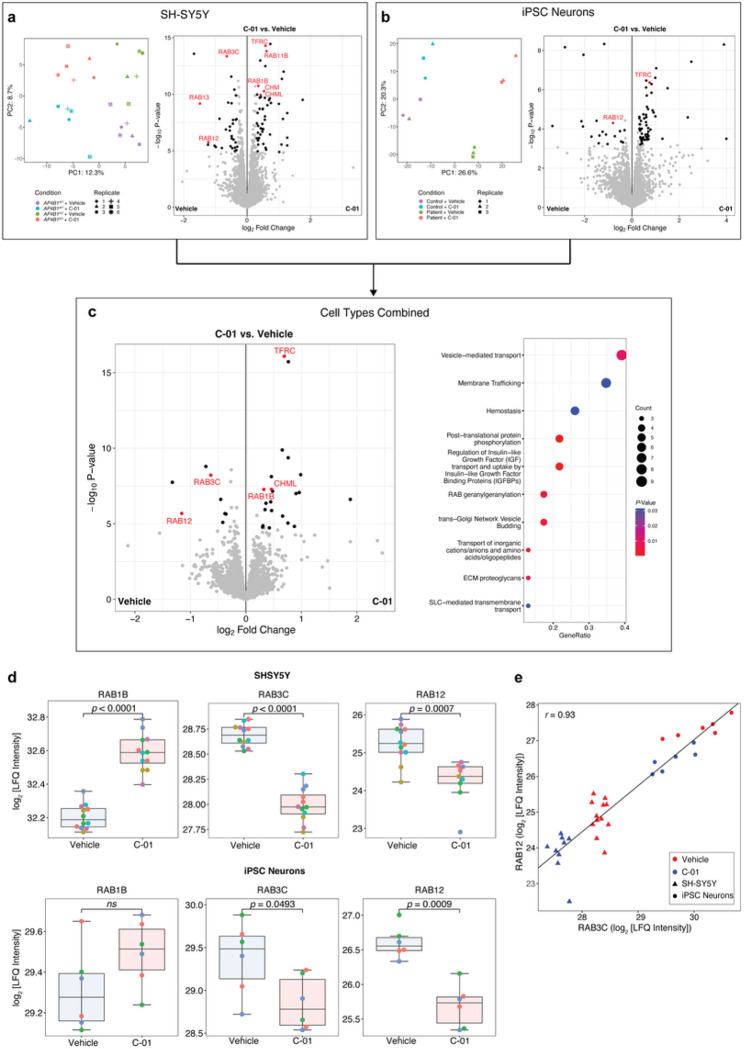
Target deconvolution using unbiased quantitative proteomics in *AP4B1*^KO^ SH-SY5Y cells and AP-4-HSP patient-derived iPSC-neurons treated with *C-01*. (a – c) Differential protein enrichment analysis. Statistical testing was done using protein-wise linear models and empirical Bayes statistics. Proteins were considered as differentially enriched with a false discovery rate of < 0.05 and a log_2_ fold change > 0.3. (a) SH-SY5Y cells: 8141 unique proteins were analyzed. PCA of the top 500 variable proteins shows robust separation between experimental conditions. The volcano plot summarizes differential protein enrichment for *AP4B1*^*WT*^ and *AP4B1*^*KO*^ cells pooled into two groups, vehicle vs. *C-01* treated. Differentially enriched proteins are depicted in black. Proteins with the most consistent enrichment profiles across all experimental conditions (see Supplementary Fig. 6a-d) are colored and labeled in red. (b) iPSC-derived neurons: 7386 unique proteins were analyzed. PCA of the top 500 variable proteins shows robust separation between experimental conditions. The volcano plot summarizes differential protein enrichment for control and patient-derived neurons pooled into two groups, vehicle vs. *C-01* treated. Differentially enriched proteins are depicted in black. Proteins with the most consistent enrichment profiles across all experimental conditions (see Supplementary Fig. 6e-h) are colored and labeled in red. (c) Integrated analysis of SH-SY5Y cells and iPSC-derived neurons: 5357 unique proteins were analyzed. The volcano plot summarizes differential protein enrichment for control and AP-4-deficient cells pooled into two groups, vehicle vs. *C-01*. Proteins with the most consistent enrichment profiles across all experimental conditions (see Supplementary Figure 6i-l) are colored and labeled in red. The dot plot summarizes dysregulated Reactome pathways of the pooled analysis. Pathways were considered differentially expressed with an FDR < 0.05. (d) The RAB protein family members RAB1B, RAB3C and RAB12 showed the most consistent profiles in response to *C-01* treatment and were selected for further analysis. LFQ intensities in SH-SY5Y cells (*AP4B1*^*WT*^ and *AP4B1*^*KO*^ pooled) and neurons (control and patient pooled) are shown. Statistical testing was done using pairwise T-tests. *P*-values were adjusted for multiple testing using the Benjamini-Hochberg procedure. (e) LFQ intensities of RAB3C and RAB12 in *AP4B1*^*WT*^ (n = 11 samples) and *AP4B1*^*KO*^ (n = 10 samples) SH-SY5Y cells, as well as control (n = 6 samples) and patient-derived (n = 6 samples) iPSC-derived neurons show a high degree of correlation measured by the Pearson correlation coefficient (*r*). While there was no difference between genotypes (not shown), *C-01* treated cells showed reduced protein levels of both RAB3C and RAB12.

To assess whether similar themes would emerge on the protein level, we next used unbiased quantitative proteomics in both differentiated SH-SY5Y cells (*AP4B1*^*KO*^ and *AP4B1*^*WT*^) and hiPSC-derived neurons (patient with *AP4B1*-associated SPG47 and control) treated for 72h with either vehicle or compound *C-01* (5μM). After quality filtering, 8,141 unique proteins in SH-SY5Y cells and 7,386 unique proteins in hiPSC-derived neurons were quantified. Differential enrichment analyses for both cell lines are shown in Fig. 7a,b, and source data are provided in Supplementary File 10. As expected, baseline quantification of differentially expressed proteins in *AP4B1*^*KO*^ SH-SY5Y cells showed downregulation of AP-4 subunits, AP4B1, AP4E1 and AP4M1, and increased ATG9A levels, as reported in other models of AP-4 deficiency ^11, 12^, ^13^ (Supplementary Fig. 6a). PCA analysis of SH-SY5Y cells demonstrated 4 distinct clusters separated by *C-01* treatment (PC1, explaining 12.3% of variance) and genotype (PC2, explaining 8.7% of variance) (Fig. 7a). Testing of vehicle vs. *C-01* treated cells showed broadly similar groups of dysregulated proteins in *AP4B1*^*WT*^ and *AP4B1*^*KO*^ SH-SY5Y cells (Supplementary Fig. 6b-d), suggesting a conserved mechanism of action independent of genotype, which allowed the pooling of cell lines to increase the power of the analysis (Fig. 7a). Similar observations were made for hiPSC-derived neurons (Fig. 7b and Supplementary Fig. 6e-h). Here, cell lines were a stronger discriminator, likely due to heterogeneity of the positive and negative controls, as expected in cell lines derived from different individuals. Again, differentially enriched proteins following *C-01* treatment in hiPSC-neurons showed a high degree of similarity between patient and control lines (Supplementary Fig. 6f-h), allowing a combined analysis (Fig. 7b).

Despite the heterogeneity in the neuronal samples, significant overlap was observed between the differentially enriched proteins in SH-SY5Y cells and hiPSC-derived neurons. Data sets were thus integrated for a combined analysis, which detected several proteins that were dysregulated across all cell types and genotypes (Supplementary Fig. 6i-l), providing strong evidence that these changes were related to treatment with *C-01* (Fig. 7c). Consistent with the overall changes in gene expression, pathway enrichment analysis using the Reactome database ^42^ highlighted engagement of intracellular trafficking pathways as a potential mechanism of action for *C-01* (Fig. 7c). Specifically, modulation of RAB proteins involved in vesicle transport emerged as a consistent theme across cell types and genotypes, with the strongest evidence for the upregulation of RAB1B and downregulation of RAB3C and RAB12. Notably, while *C-01* led to a significant change in protein levels of all three RAB protein family members in SH-SY5Y cells, only RAB3C and RAB12 reached significance in neurons (Fig. 7d). This overall pattern of RAB protein modulation was further supported by upregulation of the RAB protein geranylgeranyltransferase components A1 (CHM) in SH-SY5Y cells and A2 (CHML) in both SH-SY5Y cells and neurons. CHM and CHML play a vital role for tethering RAB proteins to intracellular membranes ^43, 44^. Additionally, upregulation of transferrin receptor protein 1 (TFRC) was observed (Fig. 7c), consistent with prior reports showing that reduction of RAB12 associates with increased protein levels of TFRC ^45^. Collectively, these findings suggest a potential role of RAB proteins in regulating vesicle transport in response to *C-01* treatment.

### RAB3C and RAB12 knockout are involved in C-01 -mediated vesicle trafficking and autophagy

RAB3C and RAB12 displayed the strongest and most consistent protein expression changes in both differentiated SH-SY5Y cells and hiPSC-derived neurons following treatment with *C-01* (Fig. 7d) and were therefore selected for further investigation. Correlation analysis revealed a strong correlation (*r* = 0.93) between the LFQ intensities of these two proteins in both cell types and across different genotypes in response to *C-01* (Fig. 7e).

To assess whether a correlation was also present on the transcriptional level, mRNA levels of *RAB3C* and *RAB12* in response to *C-01* treatment were analyzed in *AP4B1*^*WT*^ and *AP4B1*^*KO*^ SH-SY5Y cells. While there was a trend toward a reduction of *RAB3C* and elevation of *RAB12* mRNA levels and correlation analysis demonstrated a moderate inverse correlation, none of these changes reached statistical significance (Supplementary Fig. 7). These findings suggest that RAB3C and RAB12 levels are altered through a post-transcriptional mechanism following treatment with *C-01*.

**Figure 8:**
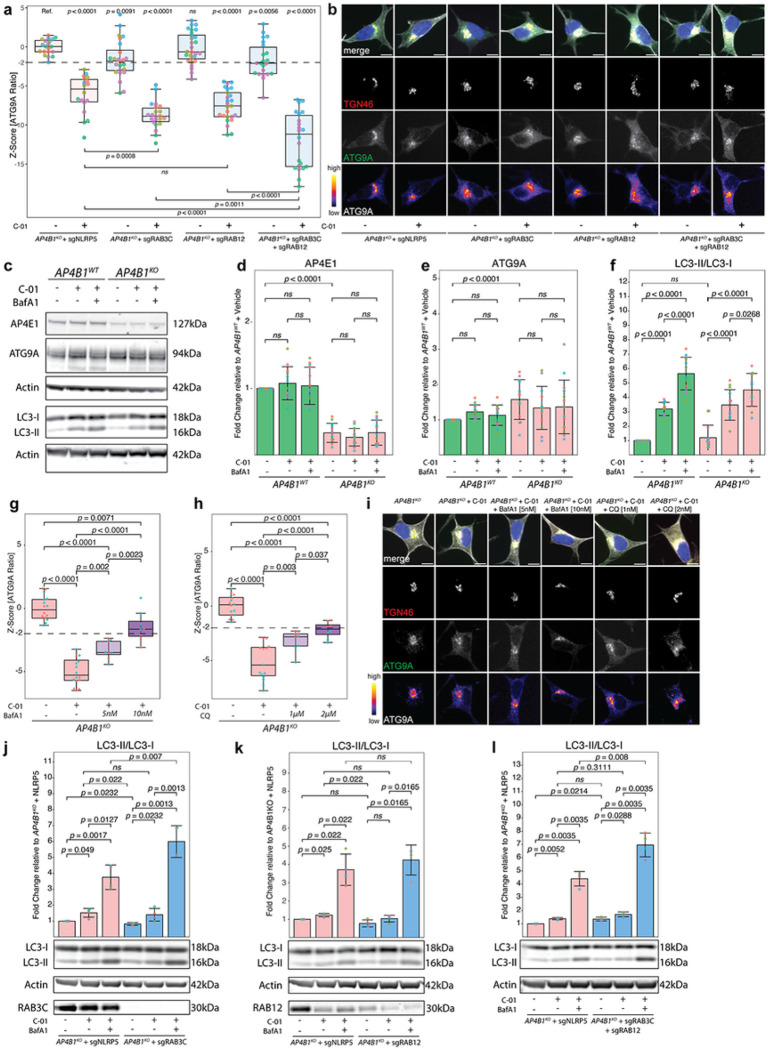
RAB3C and RAB12 are involved in *C-01*-mediated vesicle trafficking and enhancement of autophagic flux. (a) *AP4B1*^*KO*^ SH-SY5Y cells were transfected for 72h with RNPs targeting *RAB3C*, *RAB12* or both compared to *NLRP5* as a non-essential control. Vehicle vs. *C-01* treatment at a concentration of 5μM was administered for 24h. Each experimental condition was tested in 18–28 wells from 3–5 independent plates. The dashed line represents a reduction of the ATG9A ratio of −2 SD compared to the negative control (*AP4B1*^*KO*^ + sgNLRP5). Knockout of *RAB12* did not significantly alter the ATG9A ratio, while RAB3C knockout led to a reduction of −2 SD. Combining the knockout of *RAB3C* and *RAB12* did not result in an additive effect. However, both *RAB3C* and *RAB12* knockout potentiated the effect of *C-01* treatment on ATG9A translocation, which was further enhanced by combined knockout. (b) Representative images of the intracellular ATG9A distribution for different conditions. The merged image shows beta-3 tubulin (grey), DAPI (blue), the TGN (red) and ATG9A (green). The TGN and ATG9A channels are further separately depicted in greyscale. Scale bar: 10μm. (c-f) Representative western blot of whole cell lysates. Cells were treated with vehicle vs. *C-01* at a concentration of 5μM for 72h. All experiments were performed in four biological replicates. As expected, AP4E1 levels were reduced in *AP4B1*^*KO*^ cells, indicating reduced AP-4 complex formation. ATG9A ratios were significantly increased in *AP4B1*^*KO*^ cells and were not altered by *C-01* treatment. By contrast, the conversion of LC3-I to LC3-II was significantly elevated in response to *C-01* in both *AP4B1*^*WT*^ and *AP4B1*^*KO*^ cells. To confirm that this increase was due to an increase in autophagic flux, autophagosome-lysosome fusion was blocked by adding bafilomycin A1 at a concentration of 100nM for 4 h prior to cell harvest. (g-h) *AP4B1*^*KO*^ SH-SY5Y cells were treated for 72h with vehicle, *C-01* (5μM) alone, or *C-01* in combination with ascending non-toxic doses of either bafilomycin A1 (5nM or 10nM) or chloroquine (1μM or 2μM). Each experimental condition was tested in 16 wells from 2 independent plates. The dashed line represents a reduction of the ATG9A ratio of −2 SD compared to the negative control (*AP4B1*^*KO*^). As expected, *C-01* treatment alone led to a considerable reduction of the ATG9A ratio. Block of late stages of autophagy using either bafilomycin A1 or chloroquine reversed the effect of *C-01* in a dose dependent manner. (i) Representative images of the intracellular ATG9A distribution for different conditions. The merged image shows beta-3 tubulin (grey), DAPI (blue), the TGN (red) and ATG9A (green). The TGN and ATG9A channels are further separately depicted in greyscale. Scale bar: 10μm. (j-l) Western blots of whole cell lysates of *AP4B1*^*KO*^ SH-SY5Y cells transfected for 72h with RNPs against RAB3C, RAB12 or both, compared to NLRP5. Vehicle vs. *C-01* treatment was administered for 48h. While neither *RAB3C* (g) nor *RAB12* (h) knockout alone led to an increase in baseline LC3-II, the combined knockout raised the LC3-II to LC3-I ratio to levels achieved with *C-01* treatment alone (i). In response to bafilomycin A1 treatment (100nM for 4 h) both RAB3C knockout alone and the combined knockout of *RAB3C* and *RAB12* led to a significant increase in LC3-II to LC3-I ratios. Statistical testing in all experiments was done using pairwise T-tests. *P*-values were adjusted for multiple testing using the Benjamini-Hochberg procedure.

To investigate the potential impact of RAB3C and RAB12 on ATG9A translocation in the AP-4-deficient background, we used CRISPR/Cas9-mediated knockouts of RAB3C and RAB12 in *AP4B1*^*KO*^ SH-SY5Y cells (Fig. 8a,b, Supplementary Fig. 8 and Supplementary File 11). We found that knockout of RAB12 did not affect ATG9A translocation, while knockout of RAB3C caused a moderate reduction in the ATG9A ratio (Fig. 8a). Combined knockout of RAB3C and RAB12 in *AP4B1*^*KO*^ SH-SY5Y cells did not show an additive effect. Interestingly, however, the effects of *C-01* on ATG9A translocation were significantly enhanced by knockout of RAB3C, but not RAB12 alone. Combined knockout of both genes further augmented the effect of *C-01*. These findings suggest that both RAB3C alone, or in combination with RAB12, play a role in *C-01*-mediated ATG9A redistribution.

A converging theme of ATG9A translocation and alteration of RAB protein expression is autophagy. RAB proteins are known modulators of autophagy with key functions in various steps of the pathway ^46, 47^. ATG9A, a core autophagy protein, acts as a lipid scramblase and promotes autophagosome formation and elongation ^48, 49, 50, 51^. To investigate whether *C-01* leads to changes in autophagic flux, *AP4B1*^*WT*^ and *AP4B1*^*KO*^ SH-SY5Y cells were treated with *C-01* for 72h and LC3-I to LC3-II conversion was measured by western blotting (Fig. 8c–f and Supplementary Fig. 8a). Levels of LC3-II were significantly elevated in all cell lines treated with *C-01*, suggesting modulation of the autophagy pathway. Co-treatment with bafilomycin A1, which blocks autophagosome-lysosome fusion, led to further LC3-II accumulation, indicating that *C-01* increases autophagic flux (Fig. 8c–f). Blocking the late stages of the autophagy pathway, with either bafilomycin A1 or chloroquine, reversed the effect of *C-01* on ATG9A translocation in a dose-dependent manner, suggesting that this process requires intact autophagic flux (Fig. 8g–i).

Next, since our data suggested a contribution of RAB3C and RAB12 to the effect of *C-01*, we investigated the impact of RAB3C and RAB12 knockout in *AP4B1*^*KO*^ SH-SY5Y cells with and without *C-01* treatment (Fig. 8j–l and Supplementary Fig. 8b-d). Neither RAB3C nor RAB12 knockout alone led to major changes in baseline or *C-01*-enhanced autophagic flux (Fig. 8j,k). However, combined knockout of RAB3C and RAB12 significantly increased the ratio of LC3-II to LC3-I by approximately 36% (Fig. 8l). Upon treatment with bafilomycin A1, both RAB3C knockout alone and combined knockout of RAB3C and RAB12 further increased *C-01*-mediated LC3-I to LC3-II conversion (Fig. 8j–l). These findings suggest the possibility that RAB3C and RAB12 modulate *C-01*-mediated ATG9A trafficking and subsequent autophagy induction.

## DISCUSSION

Identification of novel therapeutic targets for rare neurological diseases represents a major scientific and public health challenge ^1, 4^. The increasing number of rare genetic diseases ^52^, the rising rate of diagnoses ^53^, and the significant burden for patients ^54, 55^, caregivers ^56^ and health care systems ^57^ highlight the urgent need for translational research that moves beyond gene discovery to the identification of disease mechanisms and therapies. Unbiased high-content small molecule screens are a platform for drug-repurposing approaches and a starting point for the rationale development of new compounds ^1, 2, 3, 4, 5, 6^. Disease-relevant ‘screenable’ phenotypes across cellular models, including patient-derived cells, provide an entry point into developing automated, high-content screening and analysis platforms.

In this study, we develop the first high-throughput cell-based phenotypic screening platform for a prototypical form of childhood-onset HSP caused by defective protein trafficking. Our platform allows us to determine the subcellular localization of the AP-4 cargo protein ATG9A in several cellular models of AP-4-deficiency. The hypothesis that ATG9A mislocalization is a key mechanism in the pathogenesis of AP-4-HSP is supported by the independent work of the Robinson ^12^, Kittler ^14^ and Bonifacino ^11, 13, 58^ groups, in addition to our own work ^15, 21, 24, 25^, and by the overlapping phenotypes of AP-4 ^13, 14, 26^ and *Atg9a*^28^ knockout mice.

ATG9A is the only conserved autophagy-related transmembrane protein ^50^ and in mammalian cells cycles between the TGN and ATG9A vesicles, which associate with endosomes ^59^ and autophagosome formation sites ^59, 60^. ATG9A has 4 transmembrane domains and forms homotrimers that have lipid scramblase activity ^48, 49, 50^, postulated to equilibrate lipids in the double-membrane layer of nascent autophagosomes ^61, 62^. Basal levels of autophagy are essential for neuronal survival, and neuron-specific ablation of the autophagy pathway leads to axonal degeneration and cell death ^63, 64, 65^. In neurons, autophagosomes form in the distal axon ^66, 67^ and are subject to active transport ^68, 69, 70^. Thus, efficient vesicular trafficking and spatial distribution of ATG9A are essential for axonal function as demonstrated in CNS-specific *Atg9a* knockout mice ^28^.

Having established a robust and dynamic assay that reliably measures intracellular ATG9A distribution, we systematically screened a large library of 28,864 novel small molecules for their ability to restore ATG9A trafficking from the TGN to the cytoplasm. Following this primary screen, a counter-screen and a series of orthogonal experiments identified a novel small molecule, termed *C-01*, that can restore the intracellular distribution of ATG9A and a second transmembrane AP-4 cargo protein, DAGLB, in neuronal models of AP-4 deficiency, including iPSC-derived neurons from two patients with AP-4-HSP.

Compound *C-01* has physicochemical properties that are within the parameters that are optimal for CNS drugs ^71^ and therefore represents a strong candidate for an *in vivo* tool compound. In addition, the low molecular weight and topological polar surface area create opportunities for compound optimization. Since the molecular targets of *C-01* are unknown, we employed a target deconvolution strategy using transcriptomics and proteomics to define the cellular pathways impacted by this novel small molecule. This approach identified two central themes: 1) modulation of Golgi dynamics and vesicular trafficking, and 2) engagement of autophagy. At the core of the putative pathways affected by *C-01*, we identified the Rab proteins RAB1B, RAB3C and RAB12, as well as the interacting Rab geranyl transferase subunits CHM and CHML. RAB3C and RAB12 showed the strongest and most consistent association with *C-01* treatment in both SH-SY5Y cells and iPSC-derived neurons, and our analyses suggest that these two proteins are involved in *C-01*-mediated redistribution of ATG9A from the TGN and increase of autophagic flux.

Rab proteins comprise a large family of small guanosine triphosphate (GTP) binding proteins that act as key regulators of intracellular membrane trafficking in eukaryotic cells at several stages, including cytoplasmic cargo sorting, vesicle budding, docking, fusion and membrane organization ^72, 73^. Rab GTPases function both as soluble and specifically localized, integral-membrane proteins, the latter being mediated by prenylation. Among the roughly 70 known Rab proteins, more than 20 are primarily associated with the TGN, where they regulate Golgi organization, coordinate vesicle trafficking and interact with various steps of the autophagy pathway ^46, 47^.

Following treatment with *C-01*, the RAB protein family members RAB3C and RAB12 were consistently downregulated in both SH-SY5Y cells and iPSC-derived neurons. Knockout experiments of these two proteins revealed that their loss potentiates *C-01*-mediated ATG9A translocation and autophagic flux. RAB3C, which is part of the RAB3 superfamily, is primarily expressed in brain and endocrine tissues, where it localizes to the Golgi and synaptic vesicles and is involved in exocytosis and modulation of neurotransmitter release ^74^. RAB12 is mainly localized to recycling endosomes where it regulates endosomal trafficking and lysosomal degradation and has been identified as a modulator of autophagy ^75^. A well-known downstream target of RAB12 is the transferrin receptor (TfR). Knockdown of RAB12 in mouse embryonic fibroblasts increases TfR protein levels, while overexpression leads to its reduction ^45^. In line with this, we find that treatment with *C-01* reduced RAB12 protein levels while, at the same time, robustly elevating transferrin receptor protein 1 (TFRC). To the best of our knowledge, no interaction between RAB3C and RAB12 has been described so far, however, our data suggest that both proteins are involved in *C-01*-mediated modulation of vesicle trafficking and autophagic flux.

Our study has identified the first candidate small molecule drug capable of restoring protein mislocalization in AP-4-deficient cells, including human neurons from patients. We acknowledge several limitations of our approach, some of which are inherent to high-throughput screens and some that are specific to our assay. First, as ATG9A mislocalization is a cellular phenotype of AP-4 deficiency conserved in non-neuronal and neuronal cells both *in vitro*^11, 12, 13, 14, 15, 25, 76^ and *in vivo*^13, 14, 27^, we decided to conduct the initial screen in patient-derived fibroblasts, as a simple cellular model of AP-4 deficiency. While the use of patient fibroblasts in the primary screen increases translational relevance, compounds that would have the capacity to correct ATG9A trafficking exclusively in neuronal cells could be missed at this stage. We determined that this risk was outweighed by the benefits of a robust assay performance and the fact that mechanisms of AP-4-mediated protein trafficking are conserved across tissues and cell types ^11, 12, 13, 14, 15, 35, 76^. Second, even though cell-based disease models can, to some extent, mimic the complexity of therapeutic responses in biological systems, the translation to *in vivo* models is often challenging, particularly for neurodevelopmental and neurodegenerative disease. Considerations such as a lead compound’s ability to cross the blood-brain-barrier, target engagement in the central nervous system, therapeutic responses in complex neuronal networks relying on interactions with glia cells, developmental windows amenable to therapy, as well as *in vivo* off-target effects and toxicity must be considered and explored in future studies. To mitigate some of these risks, we employed unbiased multiparametric profiling of *C-01* which suggested little off-target effects. Future studies are required to exclude pleiotropic effects or off-target toxicity in different cell types or tissues *in vivo*. Lastly, while *C-01* leads to a redistribution of two well-established AP-4 cargo proteins, ATG9A and DAGLB, we are unable to exclude the possibility that other neuron-specific cargos of AP-4 exist and are important for the pathogenesis of AP-4-HSP. Nonetheless, mislocalization of both proteins is proposed as the major contributor to neuronal pathology caused by AP-4 deficiency, through dysregulated autophagy and endocannabinoid signaling, respectively ^11, 12, 13, 14, 29^. Our automated high-throughput platform would allow for the rapid interrogation of additional AP-4 cargo proteins in the future.

In conclusion, our findings provide a solid foundation for lead optimization of *C-01* and development in Investigational New Drug (IND)-enabling studies. More broadly, our approach illustrates the development of a small molecule screening platform for a rare neurogenetic disease, leveraging robust cellular phenotypes. We hope this approach will create a paradigm for other rare and more common disorders of protein trafficking. The increase of autophagic flux through *C-01* offers the intriguing possibility that this compound could be considered for the treatment of other autophagy-associated diseases.

## METHODS

### Clinical data from patients with AP-4-HSP

This study was approved by the Institutional Review Board at Boston Children’s Hospital (IRB-P00033016 and IRB-P00016119). Two patients with AP-4-HSP and their clinically-unaffected, sex-matched parents were enrolled in the International Registry and Natural History Study for Early-Onset Hereditary Spastic Paraplegia (ClinicalTrials.gov Identifier: NCT04712812). Both patients had a clinical and molecular diagnosis of AP-4-HSP and presented with core clinical and imaging features ^8^. Patient 1 was diagnosed with *AP4B1*-associated SPG47 and carries the following compound-heterozygous variants: NM_001253852.3: c.1160_1161del (p.Thr387ArgfsTer30) / c.1345A > T (p.Arg449Ter). The sex-matched parent carries the heterozygous c.1160_1161del; p.Thr387Argfs*30 variant. Patient 2 was diagnosed with *AP4M1*-associated SPG50 and carries the following compound-heterozygous variants: NM_004722.4: c.916C > T (p.Arg306Ter) / c.694dupG (p.Glu232GlyfsTer21). The sex-matched parent carries the heterozygous c.694dupG (p.Glu232GlyfsTer21) variant.

### Antibodies and reagents

The following reagents were used: Bovine serum albumin (AmericanBIO, Cat# 9048-46-8), saponin (Sigma, #47036-50G-F), normal goat serum (Sigma-Aldrich, Cat# G9023–10ML), Dulbecco’s phosphate-buffered saline (DPBS) (Thermo Fisher Scientific, Cat# 14190–250), trypsin (Thermo Fisher Scientific, Cat#25200056), 4% paraformaldehyde (4%) (Boston BioProducts, Cat# BM-155), dimethyl-sulfoxide (DMSO) (American Bioanalytical, Cat# AB03091–00100), bafilomycin A1 (Enzo Life Sciences, Cat# BML-CM110-0100), chloroquine (MedChemExpress, Cat# HY-17589A), Molecular Probes Hoechst 33258 (Thermo Fisher Scientific, Cat# H3569) and Alexa Fluor 647-labelled phalloidin (Thermo Fisher Scientific, Cat#A22287). The following primary antibodies were used: Anti-AP4E1 at 1:500 (BD Bioscience, Cat# 612019), anti-ATG9A at 1:500–1000 (Abcam, Cat# ab108338), anti-DAGLB at 1:500 (Abcam, Cat# 191159), anti-TGN46 at 1:800 (Bio-Rad, Cat# AHP500G), anti-Golgi 97 1:500 (Abcam, Cat# 169287), anti-beta-Tubulin III 1:1000 (Synaptic Systems, Cat# 302304 and Sigma, Cat# T8660), anti-beta-Actin 1:10,000 (Sigma, Cat# A1978–100UL), pan-AKT (Cat# 4691), anti-RAB12 (Santa Cruz, Cat# sc-515613), anti-RAB3C (Santa Cruz, Cat# 107 203), anti-LC3B 1:1000 (Novus, Cat#100–2220). Fluorescently labelled secondary antibodies for immunocytochemistry were used at 1:2000 (Thermo Fisher Scientific, Cat# A11005, A-11008, A-11016, A-11073, A-21235, A-21245), for western blotting at 1:5000 (LI-COR Biosciences, Cat# 926–68022, 926–68023, 926–32212, 926–32213).

### Small molecule library

A diversity small molecule library containing 28,864 novel compounds was provided by Astellas Pharma Inc.. Compounds were arrayed in 384-well microplates at a final concentration of 10mM (1000-fold the screening concentration) in DMSO. Assay plates were stored at −80°C and thawed 30 min prior to cell plating. Active compounds from the primary screen were re-screened in a secondary screen, using eleven-point concentrations (range: 0.04μM, 0.08μM, 0.16μM, 0.31μM, 0.63μM, 1.25μM, 2.5μM, 5μM, 10μM, 20μM, 40μM) in two biological replicates. The chemical structure of the lead compound was disclosed by Astellas Pharma Inc. after the screen was completed.

### Fibroblast cell culture

Fibroblast lines were established from routine skin punch biopsies in both patients and their respective sex-matched heterozygous parents ^15^. Primary human skin fibroblasts were cultured and maintained as previously described ^77^. Briefly, cells were maintained in DMEM high glucose (Gibco, #11960044) supplemented with 20% FBS (Gibco, #10082147), penicillin 100U/ml and streptomycin 100μg/ml (Gibco, #15140122). Cells were kept in culture for up to 8 passages and routinely tested for the presence of mycoplasma contamination. For high-throughput imaging, fibroblasts were seeded onto 384-well plates (Greiner Bio-One, #781090) at a density of 2 × 10^3^ per well using the Multidrop Combi Reagent Dispenser (Thermo Fisher Scientific, #11388–558). Media changes were done every 2–3 days and drugs administered 24h before fixation.

### SH-SY5Y cell culture

*AP4B1* wildtype (*AP4B1*^*WT*^) and *AP4B1* knockout (*AP4B1*^*KO*^) SH-SY5Y cells were generated previously ^12^. Undifferentiated SH-SY5Y cells were maintained in DMEM/F12 (Gibco, Cat# 11320033) supplemented with 10% heat-inactivated fetal bovine serum (Gibco, Cat# 10438026), 100U/ml penicillin and 100μg/ml streptomycin at 37°C under 5% CO_2_. SH-SY5Y cells were passaged every 2–3 days and differentiated into a neuron-like state using a 5-day differentiation protocol with all-*trans*-retinoic acid (MedChemExpress, #HY-14649) as described previously ^33^. For assessment of ATG9A translocation, differentiated SH-SY5Y cells were plated in 96-well plates (Greiner Bio-One, Cat# 655090), at a density of 1 × 10^4^ cells per well. Media changes were done every 2–3 days and drugs administered 24–72h before fixation.

### Generation of hiPSC lines and neuronal differentiation

Fibroblasts were reprogrammed to hiPSCs using non-integrating Sendai virus as described previously ^35, 36^. Quality control experiments including karyotyping, embryoid body formation, pluripotency marker expression, STR profiling and Sanger sequencing for *AP4B1* or *AP4M1* variants were reported previously 35, 36. hiPSC-derived neurons were generated using induced NGN2 expression following published protocols with minor modifications ^37, 38^. hiPSCs were dissociated into single cells with accutase (Innovative Cell Technology, Cat#AT 104–500) and seeded onto Geltrex-coated plates (Thermo Fisher Scientific, Cat#A1413301). hiPSCs were then infected with concentrated rtTA-, and NGN2-expressing lentiviruses (FUW-M2rtTA Addgene #20342, pTet-O-Ngn2-puro Addgene #52047), in the presence of polybrene (8 μg/ml, Sigma-Aldrich, Cat# TR-1003-G). The next day, hiPSCs were fed with supplemented mTeSRPlus and expanded for cryopreservation. In parallel, a kill curve was generated to determine the optimal puromycin concentration needed to eliminate untransduced cells. Successful transduction was established by adding doxycycline (2 μg/ml, Millipore, Cat#324385–1GM) to virus-treated cells for 24 hours, followed by adding the optimized puromycin concentration (Invitrogen, 1 μg/ml, Cat# ant-pr-1) for up to 48 hours.

For the generation of glutamatergic neurons, NGN2 transduced hiPSCs were dissociated into single cells using accutase and seeded onto geltrex-coated plates. The following day, NGN2 expression was induced using doxycycline and selected with puromycin. Growth factors BDNF (10 ng/ml, Peprotech, Cat#450–02), NT3 (10 ng/ml, Peprotech, Cat# 450–03), and laminin (0.2 mg/L, Thermo Fisher Scientific, Cat#23017–015) were added in N2 medium for the first 2 days. Cells were then fed with BDNF (10 ng/ml), NT3 (10 ng/ml), laminin (0.2 mg/Lf), doxycycline (2 μg/ml), and Ara-C (4uM, Sigma-Aldrich, Cat# C1768) in B27 media every other day until differentiation day 6. On day 6, cells were dissociated with papain (Worthington, Cat# LK003178) and DNaseI (Worthington, Cat# LK003172) and replated on poly-D-lysine (0.5mg/ml; Sigma Aldrich, Cat#P6407) and laminin (5μg/ml; Thermo Fisher Scientific, Cat #23017–015) coated plates with or without hiPSC-derived astrocytes (Astro.4U, Ncardia). For assessment of ATG9A translocation, neurons were plated in 96-well plates at a density of 4 × 10^4^ cells per well. Media changes were done every 2–3 days and drugs administered 24–72h before fixation.

### Immunocytochemistry

The immunocytochemistry workflow was optimized for high-throughput using automated pipettes and reagent dispensers (Thermo Fisher Scientific Multidrop Combi Reagent Dispenser, Integra VIAFLO 96/384 liquid handler, Integra VOYAGER pipette). Fibroblasts and SH-SY5Y cells were fixed using 3% and 4% PFA, respectively, permeabilized with 0.1% saponin in PBS and blocked in 1% BSA/0.01% saponin (blocking solution) in PBS. iPSC-derived neurons were fixed in 4% PFA, and permeabilized and blocked using 0.1% Triton X-100/2% BSA/0.05% NGS in PBS. Primary antibody (diluted in blocking solution) was added for 1h (fibroblasts and SH-SY5Y cells) at room temperature or overnight (iPSC neurons) at 4°C. Plates were gently washed three times in blocking solution (fibroblasts and SH-SY5Y cells) or in PBS (iPSC neurons), followed by addition of fluorochrome-conjugated secondary antibodies, Hoechst 33258 and phalloidin for 30min (fibroblasts) or Hoechst 33258 for 60min (SH-SY5Y cells and iPSC neurons) at room temperature. Plates were then gently washed three times with PBS and protected from light.

### High-content imaging and automated image analysis

High-throughput confocal imaging was performed on the ImageXpress Micro Confocal Screening System (Molecular Devices) using an experimental pipeline modified from the pipeline described in Behne *et al.*^15^. For experiments in fibroblasts, images were acquired using a 20x S Plan Fluor objective (NA 0.45 μM, WD 8.2–6.9 mm). Per well, 4 fields were acquired in a 2×2 format (384-well plates). For experiments in SH-SY5Y cells and iPSC neurons, up to 36 fields were acquired in a 6×6 format (96-well plate) using a 40x S Plan Fluor objective ((NA 0.60 μm, WB 3.6–2.8 mm). The image analysis was performed using a customized image analysis pipeline in MetaXpress (Molecular Devices): Briefly, cells were identified based on the presence of DAPI signal inside a phalloidin (fibroblasts) or TUBB3 (SH-SY5Y cells and hiPSC-neurons)-positive cell body. Sequential masks were generated for (1) the TGN by outlining the area covered by TGN marker TGN46 (TGN46-positive area, in fibroblasts and SH-SY5Y cells) or Golgi 97 (Golgi 97-positive area, in hiPSC neurons) and (2) for the cell area outside the TGN (actin-positive area minus TGN46-positive area). ATG9A fluorescence intensity was measured in both compartments in each cell and the ATG9A ratio was calculated by dividing the ATG9A fluorescence intensity the TGN by the ATG9A fluorescence intensity in the remaining cell body (Fig. 1b):

$$ATG9ARatio=\frac{ATG9AFluoresceneIntensity(F.U.)\;\mathrm{insidetheTGN}\;}{ATG9\mathrm{AFluoresceneIntensity}\;(F.U.)\;\mathrm{outsidetheTGN}}$$

Additional masks for the TGN used for morphologic profiling included TGN Roughness (shape factor in the MetaXpress software) and the following calculated metrics:

$$\mathrm{TGNElongation}=\frac{\mathrm{TGNWith}}{\mathrm{TGNLength}}$$

$$\mathrm{TGNCompactness}=\frac{(\mathrm{TGNPerimeter}{)}^{2}}{4\pi *\mathrm{TGNArea}}$$

Z’-factor robust values and strictly standardized median difference (SSMD) ^30^ were calculated for each plate and only plates that met the predefined quality metrics of a Z’-factor robust ≥ 0.3 and SSMD ≥ 3 were included in subsequent analyses.

### Western blotting

Western blotting was done as previously described ^70^. Briefly, cells were lysed in RIPA lysis buffer (Thermo Fisher Scientific Cat# 89900) supplemented with cOmplete protease inhibitor (Roche Cat# 04693124001) and PhosSTOP phosphatase inhibitor (Roche Cat# 4906845001). Total protein concentration was determined using a Pierce BCA Protein Assay Kit (Thermo Fisher Scientific, Cat# 23225). Equal amounts of protein were solubilized in LDS sample buffer (Thermo Fisher Scientific, Cat# NP0008) under reducing conditions, separated by gel electrophoresis, using 4–12% (Thermo Fisher Scientific, Cat# NW04125BOX) or 12% Bis-Tris gels (Thermo Fisher Scientific, Cat# NP0343BOX) and MOPS or MES buffer (Thermo Fisher Scientific, #NP0001 and #NP0002) and transferred to a PVDF or nitrocellulose membranes (EMD Millipore, #SLHVR33RS). Following blocking with blocking buffer (LI-COR Biosciences, #927–70001), membranes were incubated overnight with the respective primary antibodies. Near-infrared fluorescent-labeled secondary antibodies (IR800CW, IR680LT; LI-COR Biosciences) were used and quantification was done using the Odyssey infrared imaging system and Empiria Studio Software (LI-COR Biosciences).

### Sample preparation for RNA extraction

SH-SY5Y cells were differentiated with retinoic acid as described above and subsequently treated with compounds of interest for 72h, prior to lysis using Qiagen RTL-Buffer supplemented with 1% ß-mercaptoethanol. RNA extraction, library preparation and sequencing were conducted at Azenta Life Sciences (South Plainfield, NJ, USA). Total RNA was extracted from frozen cell pellet samples using Qiagen RNeasy mini kit following manufacturer’s instructions (Qiagen, Cat# 74004).

### Library preparation with polyA selection and Illumina sequencing

RNA samples were quantified using Qubit 4 Fluorometer (Life Technologies) and RNA integrity was checked using Agilent TapeStation 4200 (Agilent Technologies). RNA sequencing libraries were prepared using the NEBNext Ultra II RNA Library Prep Kit for Illumina using manufacturer’s instructions (New England Biolabs). Briefly, mRNAs were initially enriched with Oligod(T) beads. Enriched mRNAs were fragmented for 15 minutes at 94°C. First strand and second strand cDNA were subsequently synthesized. cDNA fragments were end repaired and adenylated at 3’ends, and universal adapters were ligated to cDNA fragments, followed by index addition and library enrichment by PCR with limited cycles. The sequencing library was validated on the Agilent TapeStation (Agilent Technologies), and quantified by using Qubit 4 Fluorometer (Invitrogen) as well as by quantitative PCR (KAPA Biosystems). The sequencing libraries were clustered on 3 lanes of a flowcell. After clustering, the flowcell was loaded on the Illumina instrument (HiSeq 4000 or equivalent) according to manufacturer’s instructions. The samples were sequenced using a 2×150bp Paired End (PE) configuration. Image analysis and base calling were conducted by the Control software. Raw sequence data (.bcl files) generated the sequencer were converted into fastq files and de-multiplexed using Illumina’s bcl2fastq 2.17 software. One mismatch was allowed for index sequence identification.

### Downstream RNA sequencing analysis

Sequencing reads were mapped to the GRCh38 reference genome available on ENSEMBL using the STAR aligner v.2.7.9a. Differential expression analysis was done using the TREAT approach developed by McCarthy and Smyth ^78^, implemented in the edgeR package in R. Raw counts were obtained using STAR and low expressed genes were excluded using the method described by Chen et al. ^79^. Expression data were normalized using the Trimmed Mean of M-values method implemented in the edgeR package. Genes were considered as differentially expressed according to default options with a false discovery rate (Benjamini-Hochberg procedure) < 0.05 and a log_2_ fold change of > 0.3. Gene ontology (GO) enrichment analysis was done using clusterProfiler ^80^. Pathways were considered differentially expressed with an FDR < 0.05.

### Network connectivity analysis

To identify transcriptional changes in co-expressed groups of genes following compound treatment, a weighted gene co-expression network analysis (WGCNA) was performed.

Raw counts were generated, and low expressed genes were removed as described above. Data were normalized using variance stabilizing transformation as described by Anders *et al.*^81^. Batch effects were removed using the limma package in R ^82^. Preprocessed data were then analyzed using the WGCNA package in R ^83, 84^. In brief, pairwise Pearson correlations were calculated between all genes and genes with a positive correlation were selected to form a “directed” correlation matrix. Next, the correlations were raised to a power to approximate a scale free network. The adequate power was chosen based on soft thresholding aiming for a high scale independence above 0.8 by keeping a mean connectivity between 200 and 500. Genes were then grouped based on topological overlap and clusters were isolated using hierarchical clustering and adaptive branch pruning of the hierarchical cluster dendrogram, giving rise to groups of co-expressed genes, so called modules. Gene expression profiles within each module were summarized using the “module eigengene” (ME), defined as the first principal component of a module. Within each module, association of MEs with measured clinical traits was examined by correlation analysis. For these selected modules, module eigengene based connectivity was determined for every gene by calculating the absolute value of the Pearson correlation between the expression of the gene and the respective ME, producing a quantitative measure of module membership (MM). Similarly, the correlation of individual genes with the trait of interest was computed, defining gene significance (GS). Using the GS and MM, an intramodular analysis was performed, allowing identification of genes that have high significance with treatment as well as high connectivity to their modules. The biological information contained in modules of interest was summarized with gene ontology (GO) enrichment analysis using clusterProfiler ^80^. Pathways were considered differentially expressed with an FDR < 0.05.

### Sample preparation for mass spectrometry

Cells were lysed for whole proteome analysis in RIPA lysis buffer (Thermo Fisher Scientific, Cat# 89900) supplemented with cOmplete protease inhibitor (Roche Cat# 04693124001) and PhosSTOP phosphatase inhibitor (Roche Cat# 4906845001) and sonicated in a Bioruptor^®^ Pico Sonication System (one single 30 seconds on/off cycle at 4°C). Protein concentrations were determined using a Pierce BCA Protein Assay Kit (Thermo Fisher Scientific Cat# 23225). Lysates were stored at −80° C until further processing. To generate peptide samples for analysis by mass spectrometry, 30–50μg protein were precipitated by overnight incubation in 5 volumes of ice-cold acetone at − 20° C and pelleted by centrifugation at 10,000×g for 5 min at 4° C. All subsequent steps were performed at room temperature. Precipitated protein pellets were air-dried, resuspended for denaturation and reduction in digestion buffer (50 mM Tris pH 8.3, 8M Urea, 1 mM dithiothreitol (DTT)) and incubated for 15 min. Proteins were alkylated by addition of 5 mM iodoacetamide for 20 min in the dark. Following reduction and alkylation, proteins were enzymatically digested by addition of LysC (1 μg per 50 μg of protein; Wako, Cat# 129–02541) for an overnight incubation. Samples were then diluted four-fold with 50 mM Tris pH 8.3 before addition of Trypsin (1μg per 50μg of protein; Sigma-Aldrich, Cat# T6567) for 3 hours. The digestion reaction was stopped by addition of 1% (v/v) trifluoroacetic acid (TFA) and samples were incubated on ice for 5min to precipitate contaminants, which were pelleted by centrifugation at 10,000×g for 5min. Acidified peptides were transferred to new tubes, before purification by solid-phase extraction using poly(styrenedivinylbenzene) reverse-phase sulfonate (SDB-RPS; Sigma-Aldrich, Cat# 66886-U) StageTips ^85^. StageTips with three SDB-RPS plugs were washed with 100% acetonitrile, equilibrated with StageTip equilibration buffer (30% [v/v] methanol, 1% [v/v] TFA), and washed with 0.2% (v/v) TFA. 20μg of peptides in 1% TFA were then loaded onto the activated StageTips, washed with 100% isopropanol, and then 0.2% (v/v) TFA. Peptides were eluted in three consecutive fractions by applying a step gradient of increasing acetonitrile concentrations: 20μL SDB-RPS-1 (100 mM ammonium formate, 40% [v/v] acetonitrile, 0.5% [v/v] formic acid), then 20μL SDB-RPS-2 (150 mM ammonium formate, 60% [v/v] acetonitrile, 0.5% [v/v] formic acid), then 30μL SDB-RPS-3 (5% [v/v] NH4OH, 80% [v/v] acetonitrile). Eluted peptides were dried in a centrifugal vacuum concentrator, resuspended in Buffer A* (0.1% (v/v) TFA, 2% (v/v) acetonitrile), and stored at − 20° C until analysis by mass spectrometry.

### Mass spectrometry

Mass spectrometry was performed on an Exploris 480 mass spectrometer coupled online to an EASY-nLC 1200, via a nano-electrospray ion source (all from Thermo Fisher Scientific). Per sample, 250 ng of peptides were loaded on a 50 cm by 75μm inner diameter column, packed in-house with ReproSil-Pur C18-AQ 1.9 μm silica beads (Dr Maisch GmbH). The column was operated at 50° C using an in-house manufactured oven. Peptides were separated at a constant flow rate of 300nL/min using a linear 110min gradient employing a binary buffer system consisting of Buffer A (0.1% [v/v] formic acid) and Buffer B (80% acetonitrile, 0.1% [v/v] formic acid). The gradient ran from 5 to 30% B in 84min, followed by an increase to 60% B in 8min, a further increase to 95% B in 4min, a constant phase at 95% B for 4min, and then a washout decreasing to 5% B in 5min, before re-equilibration at 5% B for 5min. The Exploris 480 was controlled by Xcalibur software (v.4.4, Thermo Fisher Scientific) and data were acquired using a data-dependent top-15 method with a full scan range of 300–1650 Th. MS1 survey scans were acquired at 60,000 resolution, with an automatic gain control (AGC) target of 3 × 10^6^ charges and a maximum ion injection time of 25ms. Selected precursor ions were isolated in a window of 1.4 Th and fragmented by higher-energy collisional dissociation (HCD) with normalized collision energies of 30. MS2 fragment scans were performed at 15,000 resolution, with an AGC target of 1 × 10^5^ charges, a maximum injection time of 28ms, and precursor dynamic exclusion for 30s.

### Raw mass spectrometry data analysis

Mass spectrometry raw files were processed in MaxQuant Version 2.1.4.0 ^86, 87^, using the human SwissProt canonical and isoform protein database, retrieved from UniProt (2022_09_26; www.uniprot.org). Label-free quantification was performed using the MaxLFQ algorithm ^88^. Matching between runs was enabled to match between equivalent and adjacent peptide fractions, within replicates. LFQ minimum ratio count was set to 1 and default parameters were used for all other settings. All downstream analyses were performed on the ‘protein groups’ file output from MaxQuant.

### Proteomic downstream data analysis

Differential enrichment analysis of proteomics data was done using the DEP package in R. Preprocessing and quality filtering was performed separately for SH-SY5Y cells and hiPSC-derived neurons. Proteins that were only identified by a modification site, or matched the reversed part of the decoy database, as well as commonly occurring contaminants were removed. Duplicate proteins were removed based on the corresponding gene names by keeping those with the highest total MS/MS count across all samples. All following steps were done separately for each cell type (SH-SY5Y cells (Fig. 7a and Supplementary Fig. 6a-d) and hiPSC-derived neurons (Fig. 7b and Supplementary Fig. 6e-h) and for the pooled dataset (Fig. 7c and Supplementary Fig. 6i-l). Low quality entries were removed by keeping only those proteins that had valid MS/MS counts in all replicate samples of at least one experimental condition. Finally, only those proteins were kept that had a maximum of one missing LFQ value in at least one experimental condition. Filtered data were normalized using variance stabilizing transformation and missing values were imputed using a manually defined left-shifted Gaussian distribution with a width of 0.3 and a left-shift of 2.2 SD. Batch effects were corrected using the method described by Johnson *et al.*^89^. Statistical testing for differential protein enrichment was done using protein-wise linear models and empirical Bayes statistics implemented in the limma package in R. Proteins were considered as differentially enriched with a false discovery rate of < 0.05 and a log_2_ fold change > 0.3. The biological information contained in differentially enriched proteins was summarized using Reactome pathway annotation in clusterProfiler ^80^. Pathways were considered differentially expressed with an FDR < 0.05.

### Nucleofection

sgRNAs against *NLRP5*, *RAB3C* and *RAB12* were purchased as multi-guide knockout kits (v2) from Synthego, diluted to 100 μM stock concentrations and kept at −20°C. Nucleofection was performed under RNAse-free conditions on a Lonza 4D-Nucleofector (Cat# AAF-1003X, AAF-1003B) according to the manufacturer’s protocol. Briefly, SH-SY5Y cells were harvested, and 4 × 10^5^ cells resuspended in 5 μl Nucleofector Solution. 180 pmol sgRNAs were incubated with 20 pmol Cas9 protein in Nucleofector Solution to form ribonucleoprotein complexes (RNPs) according to the manufacturer’s instructions. The cell solution was then incubated with the respective RNPs and transferred into a nucleofection strip (Cat# V4XC-2032). Strips were placed in the 4D-Nucleofector System, and nucleofection was done using the CA-137 program. Following nucleofection, pre-warmed medium was added after 10 min, and cells were plated. Compound treatment was started 48h after nucleofection. Knockout efficiency of sgRNAs was assessed using the Synthego ICE Analysis online tool. Genomic DNA was extracted from nucleofected cells using the Quick-DNA Microprep Kit (Zymo Research, Cat# D3021) according to manufacturer’s instructions and amplified by PCR using the Platinum^™^ II Hot-Start PCR Master Mix (Thermo Fisher Scientific, Cat# 14000012). After a hot start, a denaturation temperature of 95° C, an annealing temperature of 58° C and an extension temperature of 72°C were chosen and repeated for 40 cycles. For amplification the following primers were used, while for sequencing only the forward primer was used: *NLRP5* forward: CTTGAGAATTTGCTGCAAGATCCT, *NLRP5* reverse: CGATTCTTCCCTGTTCCCATGAG, *RAB3C* forward: CCACTCGCCTCCTGAGTGTCTG, *RAB3C* reverse: GAACAAGGCAGAAAGTTTCTCCC, *RAB12* forward: CTGTGCGCATGGGAGTGTTTTC, *RAB12* reverse: CTTACCCACGGTGGACTTGC.

### Statistical analyses

Statistical analysis of continuous variables was performed with R version 4.2.1 (2022–06-23) and RStudio (version 2022.07.1; RStudio, Inc.) using either mean and standard deviation (SD) or median and interquartile range (IQR), depending on the distribution of data tested by visualization with histograms, quantile-quantile plots and normality testing using the Shapiro-Wilk test. Sample sizes are indicated (n) for each analysis. The T-Test (for normally distributed variables) and the Mann-Whitney U test (for non-parametric distributions) was performed to test for statistical differences.

## Data Availability

RNA sequencing data will be made publicly available through the National Center for Biotechnology Information’s Sequence Read Archive (SRA) [accession number: *pending*]. Mass spectrometry proteomics data will be deposited to the ProteomeXchange Consortium [http://proteomecentral.proteomexchange.org; *link to be updated*] via the PRIDE partner repository. Data tables with source data are provided in the supplementary material. Source images are available from the author upon reasonable request. All fibroblast and iPSC lines generated in this study are available with a material transfer agreement.

## References

[1] Tambuyzer E, Therapies for rare diseases: therapeutic modalities, progress and challenges ahead. Nat Rev Drug Discov 19, 93–111 (2020). 31836861 10.1038/s41573-019-0049-9

[2] Moffat JG, Vincent F, Lee JA, Eder J, Prunotto M. Opportunities and challenges in phenotypic drug discovery: an industry perspective. Nat Rev Drug Discov 16, 531–543 (2017). 28685762 10.1038/nrd.2017.111

[3] Eder J, Sedrani R, Wiesmann C. The discovery of first-in-class drugs: origins and evolution. Nat Rev Drug Discov 13, 577–587 (2014). 25033734 10.1038/nrd4336

[4] Sun W, Zheng W, Simeonov A. Drug discovery and development for rare genetic disorders. Am J Med Genet A 173, 2307–2322 (2017). 28731526 10.1002/ajmg.a.38326 PMC5662129

[5] Macarron R, Impact of high-throughput screening in biomedical research. Nat Rev Drug Discov 10, 188–195 (2011). 21358738 10.1038/nrd3368

[6] Vincent F, Developing predictive assays: the phenotypic screening “rule of 3”. Sci Transl Med 7, 293ps215 (2015). 10.1126/scitranslmed.aab1201 26109101

[7] Ebrahimi-Fakhari D, Behne R, Davies AK, Hirst J. AP-4-Associated Hereditary Spastic Paraplegia. In: GeneReviews((R)) (eds Adam MP, et al.) (2018). 30543385

[8] Ebrahimi-Fakhari D, Defining the clinical, molecular and imaging spectrum of adaptor protein complex 4-associated hereditary spastic paraplegia. Brain 143, 2929–2944 (2020). 32979048 10.1093/brain/awz307 PMC7780481

[9] Ebrahimi-Fakhari D, Systematic Analysis of Brain MRI Findings in Adaptor Protein Complex 4-Associated Hereditary Spastic Paraplegia. Neurology 97, e1942–e1954 (2021). 34544818 10.1212/WNL.0000000000012836 PMC8601212

[10] Ebrahimi-Fakhari D, Clinical and genetic characterization of AP4B1-associated SPG47. Am J Med Genet A 176, 311–318 (2018). 29193663 10.1002/ajmg.a.38561

[11] Mattera R, Park SY, De Pace R, Guardia CM, Bonifacino JS. AP-4 mediates export of ATG9A from the trans-Golgi network to promote autophagosome formation. Proc Natl Acad Sci U S A 114, E10697–E10706 (2017). 29180427 10.1073/pnas.1717327114 PMC5740629

[12] Davies AK, AP-4 vesicles contribute to spatial control of autophagy via RUSC-dependent peripheral delivery of ATG9A. Nat Commun 9, 3958 (2018). 30262884 10.1038/s41467-018-06172-7 PMC6160451

[13] De Pace R, Altered distribution of ATG9A and accumulation of axonal aggregates in neurons from a mouse model of AP-4 deficiency syndrome. PLoS Genet 14, e1007363 (2018). 29698489 10.1371/journal.pgen.1007363 PMC5940238

[14] Ivankovic D, Axonal autophagosome maturation defect through failure of ATG9A sorting underpins pathology in AP-4 deficiency syndrome. Autophagy 16, 391–407 (2020). 31142229 10.1080/15548627.2019.1615302 PMC6999640

[15] Behne R, Adaptor protein complex 4 deficiency: a paradigm of childhood-onset hereditary spastic paraplegia caused by defective protein trafficking. Hum Mol Genet 29, 320–334 (2020). 31915823 10.1093/hmg/ddz310 PMC7001721

[16] Dell’Angelica EC, Mullins C, Bonifacino JS. AP-4, a novel protein complex related to clathrin adaptors. J Biol Chem 274, 7278–7285 (1999). 10066790 10.1074/jbc.274.11.7278

[17] Hirst J, Bright NA, Rous B, Robinson MS. Characterization of a fourth adaptor-related protein complex. Mol Biol Cell 10, 2787–2802 (1999). 10436028 10.1091/mbc.10.8.2787 PMC25515

[18] Gadbery JE, Integrating structural and evolutionary data to interpret variation and pathogenicity in adapter protein complex 4. Protein Sci 29, 1535–1549 (2020). 32285480 10.1002/pro.3870 PMC7255511

[19] Sanger A, Hirst J, Davies AK, Robinson MS. Adaptor protein complexes and disease at a glance. J Cell Sci 132, jcs222992 (2019). 31636158 10.1242/jcs.222992

[20] Dell’Angelica EC, Bonifacino JS. Coatopathies: Genetic Disorders of Protein Coats. Annu Rev Cell Dev Biol 35, 131–168 (2019). 31399000 10.1146/annurev-cellbio-100818-125234 PMC7310445

[21] Ebrahimi-Fakhari D, High-throughput imaging of ATG9A distribution as a diagnostic functional assay for adaptor protein complex 4-associated hereditary spastic paraplegia. Brain Commun 3, fcab221 (2021). 34729478 10.1093/braincomms/fcab221 PMC8557665

[22] Ebrahimi-Fakhari D, Congenital disorders of autophagy: an emerging novel class of inborn errors of neuro-metabolism. Brain 139, 317–337 (2016). 26715604 10.1093/brain/awv371 PMC5841365

[23] Teinert J, Behne R, Wimmer M, Ebrahimi-Fakhari D. Novel insights into the clinical and molecular spectrum of congenital disorders of autophagy. J Inherit Metab Dis 43, 51–62 (2020). 30854657 10.1002/jimd.12084

[24] D’Amore A, Loss of ap4s1 in zebrafish leads to neurodevelopmental defects resembling spastic paraplegia 52. Ann Clin Transl Neurol 7, 584–589 (2020). 32216065 10.1002/acn3.51018 PMC7187712

[25] Ziegler M, Blended Phenotype of Silver-Russell Syndrome and SPG50 Caused by Maternal Isodisomy of Chromosome 7. Neurol Genet 7, e544 (2021). 33553621 10.1212/NXG.0000000000000544 PMC7862086

[26] Matsuda S, Accumulation of AMPA receptors in autophagosomes in neuronal axons lacking adaptor protein AP-4. Neuron 57, 730–745 (2008). 18341993 10.1016/j.neuron.2008.02.012

[27] Scarrott JM, Ap4b1-knockout mouse model of hereditary spastic paraplegia type 47 displays motor dysfunction, aberrant brain morphology and ATG9A mislocalization. Brain Commun 5, fcac335 (2023). 36632189 10.1093/braincomms/fcac335 PMC9825813

[28] Yamaguchi J, Atg9a deficiency causes axon-specific lesions including neuronal circuit dysgenesis. Autophagy 14, 764–777 (2018). 28513333 10.1080/15548627.2017.1314897 PMC6070006

[29] Davies AK, AP-4-mediated axonal transport controls endocannabinoid production in neurons. Nat Commun 13, 1058 (2022). 35217685 10.1038/s41467-022-28609-w PMC8881493

[30] Bray MA, Carpenter A. Advanced Assay Development Guidelines for Image-Based High Content Screening and Analysis, 2004 edn. Eli Lilly & Company and the National Center for Advancing Translational Sciences (2017). 23469374

[31] Zhang XD. Illustration of SSMD, z score, SSMD*, z* score, and t statistic for hit selection in RNAi high-throughput screens. J Biomol Screen 16, 775–785 (2011). 21515799 10.1177/1087057111405851

[32] Malo N, Hanley JA, Cerquozzi S, Pelletier J, Nadon R. Statistical practice in high-throughput screening data analysis. Nat Biotechnol 24, 167–175 (2006). 16465162 10.1038/nbt1186

[33] Kovalevich J, Langford D. Considerations for the use of SH-SY5Y neuroblastoma cells in neurobiology. Methods Mol Biol 1078, 9–21 (2013). 23975817 10.1007/978-1-62703-640-5_2 PMC5127451

[34] Rohban MH, Systematic morphological profiling of human gene and allele function via Cell Painting. Elife 6, (2017). 10.7554/eLife.24060 PMC5386591 28315521

[35] Eberhardt K, Generation and characterization of six human induced pluripotent stem cell lines (iPSC) from three families with AP4M1-associated hereditary spastic paraplegia (SPG50). Stem Cell Res 53, 102335 (2021). 34087981 10.1016/j.scr.2021.102335 PMC8824776

[36] Teinert J, Generation and characterization of six human induced pluripotent stem cell lines (iPSC) from three families with AP4B1-associated hereditary spastic paraplegia (SPG47). Stem Cell Res 40, 101575 (2019). 31525725 10.1016/j.scr.2019.101575 PMC7269118

[37] Winden KD, Biallelic Mutations in TSC2 Lead to Abnormalities Associated with Cortical Tubers in Human iPSC-Derived Neurons. J Neurosci 39, 9294–9305 (2019). 31591157 10.1523/JNEUROSCI.0642-19.2019 PMC6867816

[38] Zhang Y, Rapid single-step induction of functional neurons from human pluripotent stem cells. Neuron 78, 785–798 (2013). 23764284 10.1016/j.neuron.2013.05.029 PMC3751803

[39] Zhang B, Horvath S. A general framework for weighted gene co-expression network analysis. Stat Appl Genet Mol Biol 4, Article17 (2005). 10.2202/1544-6115.1128 16646834

[40] Winden KD, A systems level, functional genomics analysis of chronic epilepsy. PLoS One 6, e20763 (2011). 21695113 10.1371/journal.pone.0020763 PMC3114768

[41] Langfelder P, Horvath S. Eigengene networks for studying the relationships between co-expression modules. BMC Syst Biol 1, 54 (2007). 18031580 10.1186/1752-0509-1-54 PMC2267703

[42] Gillespie M, The reactome pathway knowledgebase 2022. Nucleic Acids Res 50, D687–D692 (2022). 34788843 10.1093/nar/gkab1028 PMC8689983

[43] Andres DA, cDNA cloning of component A of Rab geranylgeranyl transferase and demonstration of its role as a Rab escort protein. Cell 73, 1091–1099 (1993). 8513495 10.1016/0092-8674(93)90639-8

[44] Cremers FP, Armstrong SA, Seabra MC, Brown MS, Goldstein JL. REP-2, a Rab escort protein encoded by the choroideremia-like gene. J Biol Chem 269, 2111–2117 (1994). 8294464

[45] Matsui T, Itoh T, Fukuda M. Small GTPase Rab12 regulates constitutive degradation of transferrin receptor. Traffic 12, 1432–1443 (2011). 21718402 10.1111/j.1600-0854.2011.01240.x

[46] Lu Q, Wang PS, Yang L. Golgi-associated Rab GTPases implicated in autophagy. Cell Biosci 11, 35 (2021). 33557950 10.1186/s13578-021-00543-2 PMC7869216

[47] Liu S, Storrie B. How Rab proteins determine Golgi structure. Int Rev Cell Mol Biol 315, 1–22 (2015). 25708460 10.1016/bs.ircmb.2014.12.002 PMC4392918

[48] Matoba K, Atg9 is a lipid scramblase that mediates autophagosomal membrane expansion. Nat Struct Mol Biol 27, 1185–1193 (2020). 33106658 10.1038/s41594-020-00518-w

[49] Maeda S, Structure, lipid scrambling activity and role in autophagosome formation of ATG9A. Nat Struct Mol Biol 27, 1194–1201 (2020). 33106659 10.1038/s41594-020-00520-2 PMC7718406

[50] Guardia CM, Structure of Human ATG9A, the Only Transmembrane Protein of the Core Autophagy Machinery. Cell Rep 31, 107837 (2020). 32610138 10.1016/j.celrep.2020.107837 PMC7388177

[51] van Vliet AR, ATG9A and ATG2A form a heteromeric complex essential for autophagosome formation. Mol Cell 82, 4324–4339 e4328 (2022). 36347259 10.1016/j.molcel.2022.10.017

[52] Bamshad MJ, Nickerson DA, Chong JX. Mendelian Gene Discovery: Fast and Furious with No End in Sight. Am J Hum Genet 105, 448–455 (2019). 31491408 10.1016/j.ajhg.2019.07.011 PMC6731362

[53] Nguengang Wakap S, Estimating cumulative point prevalence of rare diseases: analysis of the Orphanet database. Eur J Hum Genet 28, 165–173 (2020). 31527858 10.1038/s41431-019-0508-0 PMC6974615

[54] Gunne E, McGarvey C, Hamilton K, Treacy E, Lambert DM, Lynch SA. A retrospective review of the contribution of rare diseases to paediatric mortality in Ireland. Orphanet J Rare Dis 15, 311 (2020). 33148291 10.1186/s13023-020-01574-7 PMC7641805

[55] Walker CE, The collective impact of rare diseases in Western Australia: an estimate using a population-based cohort. Genet Med 19, 546–552 (2017). 27657686 10.1038/gim.2016.143 PMC5440569

[56] Sandilands K, Williams A, Rylands AJ. Carer burden in rare inherited diseases: a literature review and conceptual model. Orphanet J Rare Dis 17, 428 (2022). 36494728 10.1186/s13023-022-02561-w PMC9733280

[57] Yang G, Cintina I, Pariser A, Oehrlein E, Sullivan J, Kennedy A. The national economic burden of rare disease in the United States in 2019. Orphanet J Rare Dis 17, 163 (2022). 35414039 10.1186/s13023-022-02299-5 PMC9004040

[58] Mattera R, Williamson CD, Ren X, Bonifacino JS. The FTS-Hook-FHIP (FHF) complex interacts with AP-4 to mediate perinuclear distribution of AP-4 and its cargo ATG9A. Mol Biol Cell 31, 963–979 (2020). 32073997 10.1091/mbc.E19-11-0658 PMC7185972

[59] Orsi A, Dynamic and transient interactions of Atg9 with autophagosomes, but not membrane integration, are required for autophagy. Mol Biol Cell 23, 1860–1873 (2012). 22456507 10.1091/mbc.E11-09-0746 PMC3350551

[60] Young AR, Starvation and ULK1-dependent cycling of mammalian Atg9 between the TGN and endosomes. J Cell Sci 119, 3888–3900 (2006). 16940348 10.1242/jcs.03172

[61] Orii M, Tsuji T, Ogasawara Y, Fujimoto T. Transmembrane phospholipid translocation mediated by Atg9 is involved in autophagosome formation. J Cell Biol 220, e202009194 (2021). 33439214 10.1083/jcb.202009194 PMC7809878

[62] Chumpen Ramirez S, --Atg9 interactions via its transmembrane domains are required for phagophore expansion during autophagy. Autophagy, 1–20 (2022). 10.1080/15548627.2022.2136340 PMC10241002 36354155

[63] Hara T, Suppression of basal autophagy in neural cells causes neurodegenerative disease in mice. Nature 441, 885–889 (2006). 16625204 10.1038/nature04724

[64] Komatsu M, Loss of autophagy in the central nervous system causes neurodegeneration in mice. Nature 441, 880–884 (2006). 16625205 10.1038/nature04723

[65] Komatsu M, Essential role for autophagy protein Atg7 in the maintenance of axonal homeostasis and the prevention of axonal degeneration. Proc Natl Acad Sci U S A 104, 14489–14494 (2007). 17726112 10.1073/pnas.0701311104 PMC1964831

[66] Bunge MB. Fine structure of nerve fibers and growth cones of isolated sympathetic neurons in culture. J Cell Biol 56, 713–735 (1973). 4347207 10.1083/jcb.56.3.713 PMC2108939

[67] Hollenbeck PJ. Products of endocytosis and autophagy are retrieved from axons by regulated retrograde organelle transport. J Cell Biol 121, 305–315 (1993). 7682217 10.1083/jcb.121.2.305 PMC2200099

[68] Maday S, Holzbaur EL. Autophagosome biogenesis in primary neurons follows an ordered and spatially regulated pathway. Dev Cell 30, 71–85 (2014). 25026034 10.1016/j.devcel.2014.06.001 PMC4109719

[69] Maday S, Wallace KE, Holzbaur EL. Autophagosomes initiate distally and mature during transport toward the cell soma in primary neurons. J Cell Biol 196, 407–417 (2012). 22331844 10.1083/jcb.201106120 PMC3283992

[70] Ebrahimi-Fakhari D, Impaired Mitochondrial Dynamics and Mitophagy in Neuronal Models of Tuberous Sclerosis Complex. Cell Rep 17, 1053–1070 (2016). 27760312 10.1016/j.celrep.2016.09.054 PMC5078873

[71] Wager TT, Hou X, Verhoest PR, Villalobos A. Central Nervous System Multiparameter Optimization Desirability: Application in Drug Discovery. ACS Chem Neurosci 7, 767–775 (2016). 26991242 10.1021/acschemneuro.6b00029

[72] Stenmark H. Rab GTPases as coordinators of vesicle traffic. Nat Rev Mol Cell Biol 10, 513–525 (2009). 19603039 10.1038/nrm2728

[73] Wandinger-Ness A, Zerial M. Rab proteins and the compartmentalization of the endosomal system. Cold Spring Harb Perspect Biol 6, a022616 (2014). 25341920 10.1101/cshperspect.a022616 PMC4413231

[74] Schluter OM, Schmitz F, Jahn R, Rosenmund C, Sudhof TC. A complete genetic analysis of neuronal Rab3 function. J Neurosci 24, 6629–6637 (2004). 15269275 10.1523/JNEUROSCI.1610-04.2004 PMC6729882

[75] Matsui T, Fukuda M. Rab12 regulates mTORC1 activity and autophagy through controlling the degradation of amino-acid transporter PAT4. EMBO Rep 14, 450–457 (2013). 23478338 10.1038/embor.2013.32 PMC3642374

[76] Majumder P, Edmison D, Rodger C, Patel S, Reid E, Gowrishankar S. AP-4 regulates neuronal lysosome composition, function, and transport via regulating export of critical lysosome receptor proteins at the trans-Golgi network. Mol Biol Cell 33, ar102 (2022). 35976706 10.1091/mbc.E21-09-0473 PMC9635302

[77] Ebrahimi-Fakhari D, Reduction of TMEM97 increases NPC1 protein levels and restores cholesterol trafficking in Niemann-pick type C1 disease cells. Hum Mol Genet 25, 3588–3599 (2016). 27378690 10.1093/hmg/ddw204 PMC5179952

[78] McCarthy DJ, Smyth GK. Testing significance relative to a fold-change threshold is a TREAT. Bioinformatics 25, 765–771 (2009). 19176553 10.1093/bioinformatics/btp053 PMC2654802

[79] Chen Y, Lun AT, Smyth GK. From reads to genes to pathways: differential expression analysis of RNA-Seq experiments using Rsubread and the edgeR quasi-likelihood pipeline. F1000Res 5, 1438 (2016). 27508061 10.12688/f1000research.8987.1 PMC4934518

[80] Wu T, clusterProfiler 4.0: A universal enrichment tool for interpreting omics data. Innovation (Camb) 2, 100141 (2021). 34557778 10.1016/j.xinn.2021.100141 PMC8454663

[81] Anders S, Huber W. Differential expression analysis for sequence count data. Genome Biol 11, R106 (2010). 20979621 10.1186/gb-2010-11-10-r106 PMC3218662

[82] Ritchie ME, limma powers differential expression analyses for RNA-sequencing and microarray studies. Nucleic Acids Res 43, e47 (2015). 25605792 10.1093/nar/gkv007 PMC4402510

[83] Langfelder P, Horvath S. WGCNA: an R package for weighted correlation network analysis. BMC Bioinformatics 9, 559 (2008). 19114008 10.1186/1471-2105-9-559 PMC2631488

[84] Langfelder P, Horvath S. Fast R Functions for Robust Correlations and Hierarchical Clustering. J Stat Softw 46, i11 (2012). 23050260 PMC3465711

[85] Kulak NA, Pichler G, Paron I, Nagaraj N, Mann M. Minimal, encapsulated proteomic-sample processing applied to copy-number estimation in eukaryotic cells. Nat Methods 11, 319–324 (2014). 24487582 10.1038/nmeth.2834

[86] Cox J, Mann M. MaxQuant enables high peptide identification rates, individualized p.p.b.-range mass accuracies and proteome-wide protein quantification. Nat Biotechnol 26, 1367–1372 (2008). 19029910 10.1038/nbt.1511

[87] Sinitcyn P, MaxDIA enables library-based and library-free data-independent acquisition proteomics. Nat Biotechnol 39, 1563–1573 (2021). 34239088 10.1038/s41587-021-00968-7 PMC8668435

[88] Cox J, Hein MY, Luber CA, Paron I, Nagaraj N, Mann M. Accurate proteome-wide label-free quantification by delayed normalization and maximal peptide ratio extraction, termed MaxLFQ. Mol Cell Proteomics 13, 2513–2526 (2014). 24942700 10.1074/mcp.M113.031591 PMC4159666

[89] Johnson WE, Li C, Rabinovic A. Adjusting batch effects in microarray expression data using empirical Bayes methods. Biostatistics 8, 118–127 (2007). 16632515 10.1093/biostatistics/kxj037

